# Exploring variation in research priorities generated by AI tools

**DOI:** 10.7189/jogh.16.04037

**Published:** 2026-01-30

**Authors:** John Garry, Mark Tomlinson, Maria Lohan

**Affiliations:** 1 Queen’s University Belfast, Department of Politics and International Relations, Northern Ireland, UK; 2 Stellenbosch University, Institute for Life Course Health Research, Cape Town, South Africa; 3 Queen’s University Belfast, School of Nursing and Midwifery, Northern Ireland, UK; 4 Hitotsubashi University, Hitotsubashi Institute for Advanced Studies, Tokyo, Japan

## Abstract

**Background:**

Artificial intelligence (AI) tools based on large language models (LLMs) are being increasingly used by researchers and may play a role in health-related research priority-setting exercises (RPSEs). However, little is known about how these tools may differ in the types of research priorities they generate.

**Methods:**

We examined research priorities aimed at improving treatments for four diseases: cancer, COVID-19, HIV, and Alzheimer. We compared the outputs from five AI tools (DeepSeek, ChatGPT, Claude, Perplexity, and Gemini) using SBERT-BioBERT embeddings and cosine similarity scores, and assessed the stability of differences between them by re-running identical prompts and slightly modified versions.

**Results:**

We found that the outputs produced by Gemini were highly similar to those produced by the other tools. The two most different outputs were those produced by DeepSeek and Perplexity, whereby the former tended to emphasise technical medical issues, while the latter emphasised public health concerns. This substantive distinction between DeepSeek and Perplexity remained stable across repeated and tweaked prompts.

**Conclusions:**

Our exploratory analysis suggests that Gemini performs well for researchers who prefer to generate health-related research priorities using a single AI model. For those planning to draw on multiple models, Perplexity and DeepSeek offer complementary perspectives.

Research priority-setting exercises (RPSEs) in healthcare use evidence gathered from stakeholders, such as academics and practitioners, to generate a rank-ordered list of research priorities on a particular theme [1–6]. As RPSEs become more systematic and transparent, funders can increasingly consult the produced priority lists to avoid making arbitrary or (unconsciously) biased funding decisions [7].

In general, RPSEs adopt either the Delphi method or the Child Health and Nutrition Research Initiative (CHNRI) approach [1], and consequently involve numerous rounds of question generation, synthesis and ranking [3]. Given the human resource intensiveness of these activities, and in the light of the emergence of artificial intelligence (AI) tools such as ChatGPT, DeepSeek, Gemini, and others, the question arises: can AI play a role in RPSEs? The authors of a study exploring this question by generating a ChatGPT priority listing on ageing argued that, while the results appeared compelling, they had to be closely interpreted by humans [8]. Another study focusing on global pandemic preparedness found that an AI-based RPSE can, to a significant extent, actually replicate conventional ‘gold standard’ human-generated results [9].

Advantages of using AI tools may include speed, affordability, and the inclusiveness associated with distilling priorities from the vast resource of stored human knowledge on the internet. Disadvantages may include the apparent lack of transparency of any AI-based method of generating research priorities (what exactly is happening inside the ‘black box’? [7]) and a risk of alienating human stakeholders whose engagement on the ground in actually implementing change is crucial [10,11].

When considering AI’s potential role in RPSEs, we believe it is useful to think not in terms of AI *vs*. human exercises, but rather about how the approaches may complement each other, and the conditions under which a more or less human (or AI) approach is valuable [7]. If researchers do use AI to generate research priorities (perhaps as one piece of evidence in a larger human-conducted RPSE), a pertinent question is: which particular AI tool should be used? If research priorities are generated by AI, the results should presumably not be highly sensitive to which tool is employed. If they are, and the results from just one tool are used, this may limit confidence in the results, as the results would be different if alternative tools were used.

In this paper, we explore variations in the priorities identified by five widely-used AI tools (ChatGPT, DeepSeek, Perplexity, Claude, and Gemini) for four areas of disease treatment (cancer, HIV, COVID-19, and Alzheimer’s disease). Our general departure question is: when the AI tools are asked to produce research priorities that would lead to improvement in how these diseases are treated, do they produce similar or different research priorities?

We focus on four diseases, rather than one, for the following reasons. First, we wished to examine more than a single case to avoid the risk of generalising from one, perhaps idiosyncratic, disease. Second, we wanted to set a manageable number of cases to allow for a more detailed comparison of the approaches for each case. We otherwise focused on these particular four diseases (*i.e.* cancer, COVID-19, Alzheimer’s disease, and HIV) because they are highly prevalent, include major communicable and non-communicable diseases (NCDs), and are common in both low-income and high-income countries.

According to the World Health Organization, NCDs – which include, but are not limited to, cancers, stroke, Alzheimer’s disease and cardiovascular disease – are collectively responsible for approximately 75% of all deaths worldwide [12]. Cancer is one of the leading causes of deaths from NCDs [12]. One study estimated that there were 18.1 million new cancer cases and 9.6 million cancer deaths globally in 2018 [13]. By 2040, those figures will nearly double, with the greatest increase in low- and middle-income countries (LMICs), where more than 60% of the world's cancers will occur [13]. Alzheimer’s disease (as the most prevalent form of dementia) is a major non-communicable disease and is currently ranked as the seventh leading cause of death globally [14]. In 2021, it was estimated that 57 million people across the globe have dementia, with Alzheimer’s disease accounting for 60–70% of cases [14].

Communicable diseases, including HIV and COVID-19, are also among the leading causes of death worldwide, with people living in LMICs being more likely to die of a communicable disease than an NCD [15]. Despite significant progress in tackling the disease, HIV remains a major global public health issue, with an estimated 39.3 million people living with HIV in 2023, 65% of whom were from the Africa region [16]. COVID-19, a potent example of an emerging infectious disease [17], is estimated to have led to 6.9 million recorded deaths globally since 2019 [18].

Given their global importance, these four diseases have frequently been the subject of RPSEs, albeit with varying approaches or points of focus. Specifically, RPSEs on cancer tend to focus on types of cancer such as kidney [19] or lung cancer [20], and often have a country specific [21] or regional focus [22]. In comparison, RPSEs on HIV also tend to focus on specific aspects of care or treatment, such as HIV cure [23], or HIV and mental health [24], and usually have a defined geoeconomic focus [25]. Meanwhile, RPSEs on Alzheimer’s disease have been more regularly examined under the broader umbrella of dementia and with a global lens [26], though some have also been conducted in national settings [27]. Lastly, RPSEs on COVID-19 centred less on a cure and more on management, with a keen focus across these on holistic management of impacts [28–30] and equity [31].

The aim of this paper is methodological, rather than substantive, and the four diseases are used as important illustrative cases to explore variation in the research priorities produced by the five AI tools. We suggest three specific questions about variation between AI tools that are particularly pertinent.

## In terms of generated research priorities, which AI tool is most similar to the others, and which is most dissimilar?

The answer to this question is important because researchers should be aware if the tool they are using to produce research priorities is highly idiosyncratic and likely to produce priorities that would have little overlap with priorities produced by the other tools. Researchers, conversely, may wish to know which tool is likely to be most representative, or least idiosyncratic, of all the tools. Note that we are not evaluating how ‘good’, ‘true’, ‘accurate’, or ‘valuable’ the priorities are that are produced by the different AI tools. Rather, we are simply identifying whether the tool would produce relatively unusual priorities or would instead produce ones that are typical of those generated by all of the tools.

## Which two of the tools are most dissimilar to each other, and what is the substantive nature of the difference?

If there is variation between tools, it is likely helpful for researchers to know which two particular tools are most different from each other, as choosing one rather than the other would likely lead to quite different results. Crucially, researchers will likely want to know the nature of the difference. For example, if one tool emphasises ethical research priorities and another tool generates more technical medical priorities, this can guide the researcher, depending on their aims.

Taken together, the answers to the above two questions provide a useful road map for researchers to navigate this novel terrain. Imagine that out of five AI tools, we find that tool B is the most similar to the others, while tools A and D are the most different from one another, with the former tending to emphasise technical questions and the latter emphasising ethical questions. A researcher considering the use of AI tools to generate health research priorities may decide to use a single tool and would hence perhaps opt for B as the tool that would produce the least unusual, or most representative, priorities. Alternatively, a more comprehensive strategy may be to use A, B, and D together, as this would ensure inclusion of a representative tool (B) and also ample coverage of the two themes that seem to cause most divergence among the tools: technical issues (A) and ethical (D) issues.

## How reliable are the findings?

Once differences between the priority lists generated by the five AI tools have been identified, it is important to assess the reliability of findings. Are the observed differences likely to persist if the exercise is re-run or if a slightly different prompt request to the AI tools is used? Addressing these questions enables us to understand the extent to which our findings on AI tool differences are stable or are sensitive to exactly when and how they are identified.

## METHODS

We typed the following request into each of the five AI tools: ‘Please define, and list in order of importance, the 20 greatest research priorities that would lead to improvements in the treatment of cancer/HIV/COVID-19/Alzheimer’s disease.’ We made 20 distinct requests on 9 July 2025 for four disease treatments using the following five AI tools in their default settings: ChatGPT4, Claude Sonnet 4, DeepSeek-V3, Gemini 2.5, Perplexity AI 4 (Appendix B, part A in **Online Supplementary Document**).

We measured the similarity between the priorities generated by the five AI tools as follows. For each of the top 10 priorities produced by tool A, we identified its strongest match in the top 20 of priorities of tool B. We used SBERT-BioBert (Python *via* Google Colab) to identify the strongest match as measured in terms of the highest cosine similarity score [30]. We also reversed this approach: for each of the top 10 priorities produced by tool B, we identified its strongest match in the top 20 produced by tool A. We conducted this exercise for all combinations (A/B, A/C, A/D, A/E, B/C, B/D, B/E, C/D, C/E, and D/E) and all combinations in reverse (B/A, C/A, D/A, E/A, C/B, D/B, E/B, D/C, E/C, and E/D). For each of these 20 pairings, we produced a set of the 10 strongest matches: 200 strongest matches in total for each of the four diseases, resulting in a grand total of 800 strongest matches.

To address question one (‘Which one of the five AI tools is most similar to the others, and which one of the tools is most dissimilar to the others?’), we began by distinguishing, for each disease, the following two categories: matches involving tool A *vs*. matches not involving tool A. We generated the mean cosine similarity score for each of the two categories and compared them. If the mean similarity score for matches involving tool A was higher than the mean similarity score for matches not involving tool A, we concluded that tool A is relatively good at producing priorities that overlap with the priorities produced by other tools. If the opposite finding emerged, we concluded that tool A produces quite unique priorities and has relatively low overlap with the priorities produced by the other tools. We repeated this for tools, B, C, D, and E.

To address question two (‘Which two of the tools are most dissimilar to each other, and what is the substantive nature of the difference?’), we broke down the results by each of the 20 pairings. We rank-ordered the pairings by mean cosine similarity score to identify the least similar pair. We then focused on the substantive priorities of this most dissimilar pair, identifying priorities that are unique to each tool, as measured in terms of priorities for which the strongest match is less than a cut-off point of cosine similarity score of 0.6.

To examine consistency over time and the potential effect of tweaking question prompts, we focused on the question of the substantive nature of the difference between the two most distinct tools. For both tools and all four diseases, we generated, at a subsequent time point (31 July 2025), research priorities using exactly the same prompt (Appendix B, part B in the **Online Supplementary Document**). We followed the logic described above to identify priorities unique to each tool and we interpreted the differences. We also conducted the same exercise (comparing the two most distinct tools) using a tweaked version of the question prompt, as follows (Appendix B, part C in the **Online Supplementary Document**): ‘Please imagine that you are a researcher in the area of the treatment of disease. I would like you to focus on cancer/COVID-19/Alzheimer’s disease/HIV. Please identify the 20 most important research priorities that would lead to improvements in the treatment of cancer/COVID-19/Alzheimer’s disease /HIV. Please define, and list in order of importance these 20 greatest research priorities.’

## RESULTS

### Which one of the tools is most similar to the others, and which one of the tools is most dissimilar to the others?

The mean cosine similarity scores for bilateral matching involving Gemini priorities were higher than the mean scores not involving Gemini priorities (**Table 1**). This is the case for all four diseases, with the differences for COVID-19 and cancer being statistically significant. Furthermore, the mean score across the four diseases involving Gemini was statistically significantly higher than the mean of the comparisons not involving Gemini (0.741 *vs*. 0.714; mean difference (MD) = 0.027; *P* = 0.001). The findings were similar (though less strong) for Claude, with the overall mean higher than the non-Claude mean (MD = 0.016; *P* = 0.042). In contrast, DeepSeek was most dissimilar to the other tools: cosine similarity scores involving DeepSeek were lower than for non-DeepSeek, and to a statistically significant extent in all four diseases and also for the mean of all four diseases (MD = −0.032; *P* = 0.001). This pattern emerges also, though to a lesser extent, for Perplexity (overall MD = 0.024; *P* = 0.002).

**Table 1:** Cosine similarity score differences, by tool involvement*

	Mean four diseases	Alzheimer	HIV	COVID-19	Cancer
Mean of 80 matches involving Gemini	0.741	0.704	0.744	0.714	0.804
Mean of all other 120 matches	0.714	0.681	0.721	0.678	0.776
Gemini mean − rest mean	0.027†	0.023	0.023	0.036†	0.028†
*P*-value	0.001	0.128	0.082	0.005	0.020
					
Mean of 80 matches involving Claude	0.735	0.719	0.730	0.691	0.798
Mean of all other 120 matches	0.719	0.671	0.730	0.693	0.780
Claude mean − rest mean	0.016†	0.048†	0.000	−0.002	0.018
*P*-value	0.042	0.001	0.998	0.886	0.142
					
Mean of 80 matches involving Chat	0.732	0.694	0.746	0.708	0.781
Mean of all other 120 matches	0.720	0.688	0.720	0.682	0.792
Chat mean − rest mean	0.012	0.006	0.026†	0.026†	−0.011
*P*-value	0.118	0.650	0.043	0.038	0.365
					
Mean of 80 matches involving Perplexity	0.711	0.663	0.721	0.676	0.783
Mean of all other 120 matches	0.735	0.708	0.736	0.703	0.791
Perplexity mean − rest mean	−0.024‡	−0.045‡	−0.015	−0.027‡	−0.008
*P*-value	0.002	0.002	0.245	0.033	0.492
					
Mean of 80 matches involving DeepSeek	0.706	0.671	0.710	0.673	0.771
Mean of all other 120 matches	0.738	0.703	0.743	0.706	0.798
DeepSeek mean − rest mean	−0.032‡	−0.032‡	−0.033‡	−0.033‡	−0.027‡
*P*-value	0.001	0.034	0.009	0.008	0.028

*As an example, each of the top 10 Gemini priorities for Alzheimer’s disease, was assigned a ‘best match’ among the top 20 of each of the other four tools (40 in total). Also, in reverse, among the top 20 Gemini priorities for Alzheimer’s was identified a ‘best match’ for each of the top 10 priorities using each of the four other tools (40 in all). The mean of this total of 80 matches is 0.704. The results overall suggest that Gemini is the tool most similar to the other tools and DeepSeek is the tool most dissimilar to the other tools.

†Positive mean differences that are statistically significant at the 0.05 level and indicate that the tool is relatively similar to other tools.

‡Negative mean differences that are statistically significant at 0.05 and indicate that the tool is relatively dissimilar to other tools.

### Which two of the tools are most different from each other, and what is the substantive nature of the difference?

The two most different tools were DeepSeek and Perplexity. Regarding the mean of all four diseases, we noted the lowest mean cosine similarity score for the extent to which the Perplexity top 10 were captured in the DeepSeek top 20 (**Table 2**). The second lowest similarity score was the reverse: the extent to which the DeepSeek top 10 priorities were matched in the Perplexity top 20. The two pairings (DeepSeek/Perplexity and Perplexity/DeepSeek) were the two lowest of the 20 pairings for HIV, two of the three lowest (or joint lowest) for COVID-19, two of the joint lowest for cancer and two of the three lowest for Alzheimer’s disease. In contrast, six of the top eight most similar bilateral comparisons included Gemini.

**Table 2:** Mean cosine similarity score of the highest match of each of the top 10 priorities of tool A in the top 20 of tool B*

Tool A	Tool B	Mean of four Diseases	HIV	COVID	Cancer	Alzheimer
Gemini	Claude	0.77	0.73	0.73	0.86	0.75
Gemini	Chat	0.77	0.79	0.74	0.81	0.72
Claude	Gemini	0.76	0.75	0.73	0.83	0.74
Gemini	Perplexity	0.76	0.76	0.72	0.83	0.71
DeepSeek	Chat	0.75	0.75	0.72	0.79	0.72
Chat	Claude	0.74	0.73	0.72	0.78	0.74
Perplexity	Gem	0.74	0.74	0.70	0.79	0.73
Chat	Gem	0.74	0.76	0.73	0.78	0.67
Chat	DeepSeek	0.73	0.72	0.70	0.79	0.72
Perplexity	Claude	0.73	0.71	0.69	0.81	0.72
Claude	Chat	0.73	0.74	0.69	0.77	0.72
Claude	Perplexity	0.72	0.74	0.66	0.80	0.68
Claude	DeepSeek	0.71	0.73	0.65	0.77	0.70
Perplexity	Chat	0.71	0.72	0.67	0.76	0.69
DeepSeek	Claude	0.71	0.71	0.66	0.77	0.69
Gemini	DeepSeek	0.71	0.72	0.67	0.77	0.67
DeepSeek	Gemini	0.71	0.70	0.70	0.77	0.65
Chat	Perplexity	0.70	0.75	0.70	0.76	0.58
DeepSeek	Perplexity	0.68	0.69	0.66	0.76	0.59
Perplexity	DeepSeek	0.66	0.66	0.61	0.76	0.62
Overall mean		0.73	0.73	0.69	0.79	0.69

*As an example, the 0.73 HIV score for Gemini/Claude may be interpreted as follows. For each of the top 10 HIV research priorities identified by Gemini, we used SBERT-BioBERT to identify its best match among any of the top 20 HIV priorities generated by Claude, as defined by the match with the highest cosine similarity score. This resulted in 10 best match scores, the average of which is 0.73. The table is ranked by ‘mean of four diseases’ column which averages the scores in the diseases columns. The results overall suggest that, of all possible bilateral comparisons, Gemini priorities are most closely matched by Claude priorities, and Perplexity priorities are least closely matched by DeepSeek priorities. The pattern is quite similar across all four diseases, as indicated by the inter-column correlations: HIV/COVID-19 (0.76; *P* = 0.001); HIV/cancer (0.44; *P* = 0.053); HIV/Alzheimer’s (0.43; *P* = 0.060); COVID-19/cancer (0.61; *P* = 0.004); COVID-19/Alzheimer’s (0.52; *P* = 0.019); cancer/Alzheimer’s (0.65; *P* = 0.002).

We observed the pattern (DeepSeek and Perplexity having least similarity and comparisons involving Gemini having most similarity) across the four diseases, as illustrated by the typically statistically significant inter-disease correlations (**Table 2**).

We identified the research priorities that, in a DeepSeek *vs*. Perplexity comparison, emerge as unique to each tool, in the sense that they fail to find a match in the other tool at the 0.6 cosine similarity score level (**Table 3**). DeepSeek tended to emphasise technical medical issues, while Perplexity generally emphasised issues relating to patient care and health system efficiency.

**Table 3:** Identifying tool-distinct priorities in the two most different tools (DeepSeek *vs*. Perplexity)

	Unique to DeepSeek		Unique to Perplexity	
	**Priority**	**Score**	**Priority**	**Score**
Alzheimer	APOE4-targeted therapies. Gene therapy/editing (*e.g.* APOE2 delivery, CRISPR). APOE4 conformation correctors.	0.46	Support for caregivers and families. Develop and evaluate interventions that support caregivers' health, well-being, and ability to provide care.	0.47
	Synaptic protection & repair. Boost neurotrophic factors (*e.g.* BDNF mimetics). Enhance synaptic plasticity (*e.g.* glutamate receptor modulators).	0.49	Improving quality and delivery of care. Optimise care models across settings (home, hospital, long-term care) and ensure evidence-based practices are implemented.	0.49
	Neuroinflammation modulation. Microglia-targeted therapies (*e.g.* TREM2 agonists, NLRP3 inhibitors). Anti-inflammatory repurposing (*e.g.* SARM1 inhibitors, JAK/STAT modulators).	0.54	Understanding disease mechanisms. Deepen knowledge of the biological, genetic, and environmental factors that drive Alzheimer’s and related dementias.	0.53
	Vascular contributions. Treat small vessel disease (*e.g.* blood-brain barrier repair). Link between hypertension/diabetes and AD.	0.56	Addressing drug resistance and treatment failure. Investigate why some patients do not respond to current treatments and develop strategies to overcome these barriers.	0.53
	Anti-tau therapeutics. Tau aggregation inhibitors (*e.g.* small molecules, antibodies). Tau clearance strategies (*e.g.* autophagy enhancers).	0.57		
HIV	bNAbs: enhance potency and durability of bNAbs for treatment/prevention. Overcome viral escape mutations via engineered multi-specific antibodies.	0.58	Optimising ART for all populations. Ensuring ART safety and efficacy for diverse groups, including children, adolescents, pregnant and lactating women, and people with comorbidities.	0.54
	Immune-based therapies. Restore CD4+ T-cell function (*e.g.* IL-2, PD-1 blockade). Enhance innate immunity (*e.g.* NK cell therapies).	0.58	Integration of HIV and other health services. Research on models to deliver integrated care for HIV alongside other infectious and non-infectious diseases, mental health, and reproductive health.	0.55
			Enhancing early detection and diagnosis. Developing and deploying sensitive, specific, and accessible diagnostic tools, including point-of-care and at-home viral load monitoring.	0.59
COVID-19	mAbs for emerging variants. Engineer variant-proof mAbs (*e.g.* bispecific antibodies). Improve delivery (*e.g.* inhaled mAbs for early infection).	0.52	Integrating COVID-19 care with other health services. Researching models for integrating COVID-19 treatment with other essential health services, especially in resource-limited settings.	0.43
	Repurposed drugs with rigorous evidence. Validate promising candidates (*e.g.* fluvoxamine, bromhexine) in large trials. Clarify roles of anticoagulants/statins in acute/post-COVID care.	0.59	Health systems strengthening and preparedness. Enhancing healthcare capacity, resource allocation, and preparedness for current and future waves or pandemics.	0.46
			Understanding and managing COVID-19 variants. Investigating the evolution, transmission, and clinical impact of SARS-CoV-2 variants to inform treatment and prevention strategies.	0.54
			Optimising clinical management across populations. Tailoring treatment protocols for diverse groups, including children, elderly, pregnant individuals, and those with comorbidities.	0.59

AD – Alzheimer’s disease, APOE – apolipoprotein E, ART – antiretroviral therapy, BDNF – brain derived neurotrophic factor, bNAbs – broadly neutralising antibodies, CD4+ – cluster of differentiation 4, positive, CRISPR – clustered regularly interspaced short palindromic repeats, IL-2 – interleukin-2, JAK/STAT – Janus kinase/signal transducer and activator of transcription, mAbs – monoclonal antibodies, NK – natural killer, PD-1 – programmed death-1, SARM1 – sterile alpha and TIR motif-containing protein 1, SARS-Cov-2 – severe acute respiratory syndrome coronavirus 2, TREM2 – triggering receptor expressed on myeloid cells 2

*A priority ‘unique’ to a tool is a priority in the top 10 produced by that tool for which a match in the top 20 in the other tool is not identified at the cosine similarity score level of 0.6. All priorities failing to reach this threshold are reported. Note that none of the cancer priorities fell below this threshold.

### Are the results the same over time and over tweaked question prompt?

The substantive differences between DeepSeek and Perplexity reported above also emerge when we generate and analyse research priorities at a second time point (Table S1 in the **Online Supplementary Document**). In a distinct analysis, we observed similar differences between DeepSeek and Perplexity when we used the tweaked version of the question prompt, as described above (Table S2 in the **Online Supplementary Document**). Across all three analyses (**Table 3**; Tables S1–3 in the **Online Supplementary Document**), Perplexity emphasised public health concerns: the importance of environmental factors; patient safety; mental and psychological health of patients and/or caregivers; the accessibility of diagnostic tools; the importance of low-income and low-resource contexts; improving healthcare quality and access and reducing health inequalities; and including diverse groups and populations. Across all three analyses, DeepSeek tended to emphasise relatively technical and detailed medical research matters.

## DISCUSSION

Our aim in this exploratory paper was not to pass judgment on whether researchers should use AI tools to identify research priorities, nor to recommend how much weight should (or should not) be attached to AI-generated priorities. Rather, our goal was to help researchers cautiously navigate this rapidly emerging and dynamic terrain. By focusing on variation in the research priorities generated by different AI tools, we highlight the potential implications of tool selection in RPSEs that incorporate large language models (LLMs).

Our findings suggest that if only a single AI tool is to be used, there are advantages to selecting Google Gemini: among the five tools we examined, it produced the least idiosyncratic results, thus giving researchers greater confidence that its outputs are more likely to align with those of other tools. Alternatively, for researchers wishing to use multiple tools, our results suggest also incorporating DeepSeek and Perplexity. This combination would likely maximise the capture of both technical medical priorities (DeepSeek) and public health-oriented priorities (Perplexity). Researchers who do opt to use multiple tools face the challenge of how to present the generated results: as distinct sets of priorities or as a single collated rank-ordered list? If the latter, a defence of how exactly the listing was ordered would have to be elaborated. For example, should priorities unique to one tool be afforded greater or lesser priority than priorities that occur across multiple tools?

We stress that the recommendations given here are provisional and we advocate for further confirmatory research. Future studies could examine a wider range of AI tools, analyse outputs across more time points to better assess stability, test a more nuanced set of prompts, and evaluate whether the patterns identified here persist as new versions of the LLMs are released. Given the rapid pace of AI development [32], such follow-up is essential.

A broader question is whether variation in LLM outputs will increase or decrease as the industry evolves. One scenario is convergence: shared regulatory standards and safeguards could push major global models in the same direction, reducing differences over time. Another is divergence: new entrants may emerge that reflect particular cultural or political contexts, producing distinct priorities – for example, Allam 34B, developed in Saudi Arabia and aligned with Islamic culture and values [33].

Our study of variation across LLMs is only one component of a larger research programme on the role of AI in RPSEs. A central issue remains the quality (or validity) of AI-generated priorities. More research is needed to examine the extent to which AI replicates human-generated priorities [9] and investigating how domain experts assess the quality of AI outputs [8]. If such validity tests were passed, it may be defensible to explore integrating AI generated priorities as a component part of established RPSEs, including the Delphi, CHNRI, or James Lind approaches.

Transparency is another crucial consideration. Opening the ‘black box’ of how AI produces research priorities must be a priority in itself. Much of the priority-setting literature of the past 25 years arose as a reaction against opaque processes in which powerful academics – often a small group of men in London or New York – set priorities behind closed doors [34,35]. Avoiding a regression to similar opacity in AI-driven processes is essential. We encourage focused research into how the outputs of AI tools may reflect geographic, disciplinary, or socioeconomic biases in their underlying LLM training data. We also encourage work clarifying of the content of the training data used to generate the LLMs to assess the extent to which AI tools are (to varying degrees) synthesising from existing RPSEs or independently generating novel priorities.

## CONCLUSIONS

Our main contribution in this paper is to propose an approach for examining variation in research priorities generated by different LLMs and to offer tentative advice on the implications of LLM selection for research priority setting exercises.

## Additional material


Online Supplementary Document
