# Supplementary material for: Exploring variation in research priorities generated by AI tools
Source: J Glob Health. 2026 Jan 30;16:04037. doi: 10.7189/jogh.16.04037 (PMC12856382; doi:10.7189/jogh.16.04037)
Supplement: Online Supplementary Document [file jogh-16-04037-s001.pdf]

Supplement to: Garry J, Tomlinson M, Lohan M. Exploring variation in research priorities generated by AI tools. J Glob Health. 2026;16:04037.

Appendix A.

(Tables S1–3)

Table S1.<br>Identifying tool-distinct priorities in the two most different tools (DeepSeek vs. Perplexity): Using the same prompts at Time 2				
	**Unique to DeepSeek**		**Unique to Perplexity**	
	**priority**	**score**	**priority**	**score**
Alzheimer's	APOE4 Therapeutics. Develop APOE4-to-APOE2/3 converters, gene therapy, or small molecules to mitigate APOE4's toxic effects.	.52	Elucidating Mechanisms of Disease Pathogenesis. Deepen understanding of the cellular, genetic, and environmental mechanisms driving Alzheimer's, including the exposome and gene-environment interactions.	.48
	Neuroinflammation Modulation. Target microglial dysfunction (e.g., TREM2, NLRP3 inflammasome) to reduce neuronal damage.	.53	Clinical Trials Innovation. Use real-world data, adaptive trial designs, and decentralized research platforms to accelerate evaluation of new interventions, including those repurposed from other conditions.	.55
	Synaptic Repair & Neuroprotection. Boost BDNF, NMDA receptor modulators, and mitochondrial function to preserve cognition.	.55		
HIV	Gene Therapy & CRISPR. Edit CCR5/CXCR4 or excise HIV provirus using gene-editing tools (e.g., EBT-101 trial).	.51	Long-term Safety and Toxicity Research. Investigating the long-term effects, side effects, and toxicity of ART, especially newer agents and long-acting formulations.	.46
	Broadly Neutralizing Antibodies (bNAbs). Develop next-gen bNAbs (e.g., VRC07-523LS) for long-acting prevention/treatment and reservoir targeting.	52	Minimizing Stigma and Improving Quality of Life. Reducing stigma, discrimination, and mental health burdens to facilitate ART adherence, retention, and holistic well-being.	.47
		.	Simplifying and Scaling Diagnostic Tools. Innovating rapid, affordable, and point-of-care diagnostics for viral load, resistance, and early infection suitable for low-resource settings.	.55
COVID-19	Antiviral Resistance Monitoring. Track emerging resistance to Paxlovid (nirmatrelvir), remdesivir, and molnupiravir.	.42	Health System Strengthening and Scalability. Optimize models of scalable acute and post-acute (long COVID) care, triage, critical care capacity, and workforce readiness.	.43
	Immunomodulators for Severe COVID. Refine timing/dosing of steroids, JAK inhibitors, IL-6 blockers to curb cytokine storms without immunosuppression.	.52	Non-Pharmacological Symptom Management. Test and optimize rehabilitation, physical therapy, dietary interventions, and psychological support strategies.	.52
	Repurposed Drug Screening. Systematically test existing drugs (e.g., metformin, fluvoxamine) for antiviral/anti-inflammatory effects.	.54	Immunity and Immune Response Research. Clarify natural and vaccine-induced immunity duration, immune correlates of protection, and immune dysfunction in long COVID.	.57
	Monoclonal Antibodies (mAbs) for Emerging Variants. Engineer broadly neutralizing mAbs resistant to viral escape mutations.	.55		
	Broad-Spectrum Antivirals. Develop pan-coronavirus drugs effective against SARS-CoV-2 variants and future outbreaks.	.57		
cancer	Microbiome & Cancer Therapy. Harness gut microbiota to improve immunotherapy response and reduce toxicity.	.56	Affordable and Equitable Access to Cancer Therapies. Address disparities in access to care by developing cost-effective treatments and health policies to make advanced therapies available globally, especially in low- and middle-income countries.	.57
	Cancer Stem Cell Eradication. Target self-renewing, therapy-resistant subpopulations driving recurrence.	.59		

**Table S2.**<br>**Identifying tool-distinct priorities in the two most different tools (DeepSeek vs. Perplexity): Using a tweaked prompt**				
	**Unique to DeepSeek**		**Unique to Perplexity**	
	**priority**	**score**	**priority**	**score**
Alzheimer’s	Blood-Brain Barrier (BBB) Penetration Strategies. Why? Many promising drugs fail due to poor BBB crossing; nanoparticle delivery or ultrasound techniques could improve efficacy.	.49	Addressing Health Disparities and Inclusion of Diverse Populations. Investigate differences in AD risk, progression, and treatment response across ethnic, racial, and socioeconomic groups to improve equity in care and research.	.46
	APOE4 & Genetic Risk Factors (Therapeutic Targeting). Why? APOE4 is the strongest genetic risk factor research into gene editing, lipid metabolism drugs, or APOE2-boosting therapies is vital.	.52	Advancement in Neuroimaging Technologies. Develop high-resolution, accessible imaging methods for better diagnosis, monitoring, and understanding of disease progression.	.48
			Development of Non-Pharmacological Interventions. Research effective cognitive, behavioral, and psychosocial therapies to improve quality of life for patients and caregivers.	.56
			Understanding Genetic and Molecular Mechanisms. Elucidate the underlying genetic, molecular, and cellular pathways driving AD onset and progression, including gene-environment interactions.	.57
HIV	Pediatric HIV Cure & Better Treatments. Why? Children face unique challenges, including drug formulations and lifelong ART burdens.	.57	Advancing Cross-Cutting Scientific Research. Integrating basic, behavioral, social, epidemiological, and implementation sciences to improve prevention, treatment, and care.	.45
			Improving HIV Diagnostics and Point-of-Care Testing. Enhanced diagnostics suitable for low- and middle-income settings, enabling rapid, accessible HIV testing and monitoring.	.49
			Pediatric and Adolescent HIV Treatment Research. Development of age-appropriate formulations and ensuring safety and efficacy data for children and adolescents.	.57
COVID-19	Next-Gen Monoclonal Antibodies (Resistant to Variants). Why? Many antibody therapies lose efficacy against new variants; engineered or multi-target antibodies could remain effective.	.50	Addressing Health Inequities. Strategies to ensure equitable access to prevention, diagnosis, and treatment worldwide, especially in low-resource settings.	.57
	Nasal & Mucosal Vaccines (To Block Transmission). Why? Current vaccines reduce severe disease but don't fully prevent infection; mucosal immunity could stop transmission.	.52	Optimization of Clinical Management Protocols. Refinement of treatment guidelines for different COVID-19 severities and patient populations based on emerging evidence.	.58

**Table S3.<br>Identifying the similarities of themes relating to Perplexity in Figures 1-3**			
Summary of similarities	***From Figure 1***	***From Figure 2***	***From Figure 3***
Environment	Environmental factors	Environmental mechanisms /<br>Gene-environment interactions	Gene-environment interactions
Safety	Safety	Long-term Safety and Toxicity Research	Safety
Mental health	Mental health /<br>Support for Caregivers and Families	Reducing stigma, discrimination, and mental health burdens /<br>Rehabilitation, physical therapy, dietary interventions, and psychological support	Behavioral, and psychosocial therapies to improve quality of life for patients and caregivers.
Accessible diagnostics	Accessible diagnostic tools	Innovating rapid, affordable, and point-of-care diagnostics	Accessible imaging methods for better diagnosis /<br>Enhanced diagnostics suitable for low- and middle-income settings,
Low-income contexts	Resource-limited settings	Low-resource settings /<br>Low- and middle-income countries.	Low-resource settings/<br>Low- and middle-income settings
Quality and fairness	Improving Quality and Delivery of Care	Affordable and Equitable Access to Cancer Therapies	Addressing Health Disparities /<br>Addressing Health Inequities
Methods		Clinical Trials Innovation.	Integrating basic, behavioral, social, epidemiological, and implementation sciences
Diverse groups	Tailoring treatment protocols for diverse groups /<br>Efficacy for diverse groups		Refinement of treatment guidelines for different COVID-19 severities and patient populations<br>Inclusion of Diverse Populations
Health system	Integrated care /<br>Integrating COVID-19 Care with Other Health Services /<br>Health Systems Strengthening and Preparedness.	Health System Strengthening and Scalability.	

Appendix B.

The generated priorities: Parts A-C

Contents

Part A: Texts for main analysis

A1: Alzheimer’s disease

A2: Cancer

A3: COVID-19

A4: HIV

Part B: Texts for over time analysis

B1: Alzheimer’s disease

B2: Cancer

B3: COVID-19

B4: HIV

Part C: Texts for tweaked prompt analysis

C1: Alzheimer’s disease

C2: Cancer

C3: COVID-19

C4: HIV

Part A: Texts for main analysis

A1: Alzheimer's disease

ChatGPT 1-20 are as follows:

1. Disease-Modifying Therapies Targeting Amyloid-β (Aβ). Goal: Develop and refine treatments that reduce Aβ plaques or production. **Why it matters**: Aβ aggregation is a hallmark of Alzheimer's pathology. **Example**: Anti-Aβ monoclonal antibodies (e.g., lecanemab, donanemab), BACE inhibitors.

2. Tau-Targeted Therapies. Goal: Inhibit tau phosphorylation, aggregation, or spread between neurons. **Why it matters**: Tau tangles correlate more closely with cognitive decline than Aβ. **Example**: Anti-tau antibodies, tau aggregation inhibitors, antisense oligonucleotides (ASOs).

3. Early Detection and Preclinical Diagnosis Tools. Goal: Identify AD before symptoms using biomarkers or imaging. **Why it matters**: Earlier treatment may prevent irreversible damage. **Example**: PET imaging, plasma phosphorylated tau (p-tau217/181), CSF biomarkers.

4. Neuroinflammation Modulation. Goal: Target glial activation and chronic inflammation in the brain. **Why it matters**: Inflammatory response contributes to neurodegeneration. **Example**: TREM2 modulation, NSAIDs, microglial-targeted therapies.

5. Neuroprotection and Synaptic Resilience. Goal: Preserve or restore synaptic function and neuronal survival. **Why it matters**: Synaptic loss directly correlates with cognitive impairment. **Example**: BDNF mimetics, NMDA receptor modulators, neurotrophic gene therapy.

6. Personalized and Precision Medicine Approaches. Goal: Tailor treatment based on genetic, biomarker, and lifestyle profiles. **Why it matters**: AD is heterogeneous; one-size-fits-all treatments fail many. **Example**: APOE4-stratified trials, polygenic risk scores, pharmacogenomics.

7. Vascular Contributions to Cognitive Impairment and Dementia (VCID). Goal: Understand and treat vascular factors contributing to AD. **Why it matters**: Many patients have mixed vascular and Alzheimer's pathology. **Example**: Blood-brain barrier integrity, antihypertensives, anti-atherosclerotics.

8. Mitochondrial Function and Oxidative Stress Reduction. Goal: Improve energy metabolism and reduce neuronal oxidative damage. **Why it matters**: Mitochondrial dysfunction exacerbates neurodegeneration. **Example**: NAD+ boosters, antioxidants, metabolic enhancers.

9. Glucose Metabolism and Insulin Resistance in the Brain. Goal: Improve brain insulin signaling to combat "type 3 diabetes" hypothesis. **Why it matters**: Insulin resistance may drive or worsen AD pathology. **Example**: Intranasal insulin, GLP-1 agonists (e.g., semaglutide), metformin.

10. Brain-Computer Interfaces and Neuromodulation. Goal: Use non-invasive stimulation to enhance cognition or slow decline. **Why it matters**: May improve symptoms and neuroplasticity even in later stages. **Example**: Transcranial magnetic stimulation (TMS), deep brain stimulation (DBS), tDCS.

11. Development of Non-Amyloid, Non-Tau Disease Targets. Goal: Explore alternative pathways: lipid metabolism, lysosomal dysfunction, etc. **Why it matters**: Aβ and tau targeting alone have shown limited success. **Example**: Progranulin enhancers, GBA-targeted drugs, autophagy inducers.

12. Sex Differences and Hormonal Influences in Alzheimer's. Goal: Understand why women are disproportionately affected and tailor treatment. **Why it matters**: Estrogen, menopause, and X-linked factors play roles. **Example**: HRT timing studies, sex-specific therapeutics.

13. Lifestyle and Multimodal Intervention Trials. Goal: Test combinations of diet, exercise, cognitive training, and vascular risk reduction. **Why it matters**: May delay onset or slow progression in at-risk individuals. **Example**: FINGER study model, multidomain prevention trials.

14. Enhancing Blood-Brain Barrier (BBB) Integrity. Goal: Protect or repair the BBB to reduce neurotoxin and immune cell infiltration. **Why it matters**: BBB dysfunction worsens neuroinflammation and AD pathology. **Example**: Targeting pericytes, MMPs, or endothelial cells.

15. Artificial Intelligence for Drug Discovery and Trial Optimization. Goal: Use AI to predict drug candidates and identify treatment-responsive subgroups. **Why it matters**: Accelerates innovation and reduces failed trials. **Example**: AI in virtual screening, adaptive trial designs.

16. Novel Symptomatic Treatments for Cognitive and Neuropsychiatric Symptoms. Goal: Improve quality of life through better control of agitation, apathy, psychosis, and memory loss. **Why it matters**: Many patients and caregivers struggle with behavioral symptoms. **Example**: Pimavanserin for psychosis, cannabinoids, digital cognitive therapies.

17. Cross-Disease Research: Alzheimer's and Other Neurodegenerative Diseases. Goal: Explore shared pathways (e.g., TDP-43, alpha-synuclein) with Parkinson's, FTD, etc. **Why it matters**: May lead to broader therapeutic targets and better classification of mixed dementias.

18. Enhancing Recruitment and Diversity in Clinical Trials. Goal: Ensure underrepresented groups (ethnic, socioeconomic) are included in research. **Why it matters**: Treatments must be generalizable and equitable. **Example**: Community engagement, culturally tailored outreach, decentralized trials.

19. Digital Biomarkers and Remote Monitoring Technologies. Goal: Track disease progression and treatment effects continuously and non-invasively. **Why it matters**: Enables early intervention and adaptive therapy. **Example**: Wearables, speech/language biomarkers, passive sensor data.

20. Support for Caregiver-Centered Interventions. Goal: Develop tools and therapies that support caregivers' mental and physical health. **Why it matters**: Caregiver burnout can accelerate patient institutionalization. **Example**: Digital respite platforms, behavioral coaching, caregiver stress therapies.

Claude 1-20 are as follows

1. Disease-Modifying Therapies Beyond Amyloid While lecanemab (Leqembi®) and donanemab (KisunlaTM) change the underlying biology of Alzheimer's disease and delay disease progression developing treatments that target multiple pathways simultaneously is crucial for meaningful therapeutic impact.

2. Tau-Targeting Therapeutics Tau has received increasing attention as a potential therapeutic target, since evidence indicates that tau pathology is more associated with cognitive dysfunction. Research into tau aggregation inhibitors and anti-tau antibodies represents a critical frontier.

3. Early Detection and Diagnostic Biomarkers The Alzheimer's Association is preparing guidelines on blood-based biomarker tests (anticipated in 2025), which could revolutionize early diagnosis and enable intervention before significant cognitive decline.

4. Neuroinflammation Modulation Inflammation, especially neuroinflammation, accompanies AD pathological processes and is also linked to cognitive deficits. Targeting microglial activation and inflammatory cascades offers therapeutic potential.

5. Combination Therapy Development Given the multifactorial nature of Alzheimer's disease, developing rational combinations of drugs targeting different pathways (amyloid, tau, inflammation, neuroprotection) is essential for enhanced efficacy.

6. Precision Medicine Approaches Creating personalized treatment strategies based on genetic profiles, biomarker signatures, and disease stage to optimize therapeutic outcomes for individual patients.

7. Neuroprotective Strategies Developing therapies that protect neurons from damage, enhance synaptic function, and promote neuroplasticity to preserve cognitive function regardless of underlying pathology.

8. Blood-Brain Barrier Enhancement Improving drug delivery to the brain through novel delivery systems, blood-brain barrier modulation, and targeted transport mechanisms to enhance therapeutic efficacy.

9. Prevention in At-Risk Populations Developing interventions for individuals with genetic risk factors or early biomarker changes to prevent or delay disease onset in asymptomatic stages.

10. Cognitive Enhancement and Symptomatic Treatment Advancing treatments that can improve cognitive function, daily living activities, and quality of life even in the presence of pathological changes.

11. Synaptic Restoration Therapies Researching methods to restore synaptic connections, enhance neurotransmitter function, and rebuild neural networks damaged by disease progression.

12. Microglial Modulation Burgeoning research points to the brain's resident immune cells, microglia, as critical players in Alzheimer's pathogenesis, making them important therapeutic targets.

13. Autophagy and Cellular Clearance Enhancing the brain's natural clearance mechanisms to remove toxic protein aggregates and damaged cellular components that contribute to disease progression.

14. Vascular Factors and Brain Health Addressing cardiovascular risk factors, improving cerebral blood flow, and targeting the vascular contributions to cognitive impairment and dementia.

15. Sleep and Circadian Rhythm Restoration Developing interventions to improve sleep quality and restore circadian rhythms, which are disrupted in Alzheimer's disease and may contribute to pathology.

16. Metabolic and Mitochondrial Therapies Targeting brain metabolism, mitochondrial function, and energy production to address the metabolic dysfunction associated with Alzheimer's disease.

17. Gut-Brain Axis Interventions Researching the role of the microbiome in Alzheimer's disease and developing interventions that target the gut-brain axis to influence neuroinflammation and cognition.

18. Advanced Clinical Trial Design Improving clinical trial methodologies, endpoint selection, and patient stratification to accelerate drug development and increase success rates.

19. Digital Therapeutics and Monitoring Developing digital tools for cognitive training, remote monitoring, and personalized interventions that can complement pharmacological treatments.

20. Caregiver Support and Healthcare Delivery Researching optimal models of care delivery, caregiver support systems, and healthcare infrastructure to improve patient and family outcomes.

DeepSeek 1-20 are as follows:

1. Early Detection & Biomarkers. **Blood-based biomarkers** (e.g., p-tau217, GFAP) for pre-symptomatic diagnosis. **Cost-effective screening tools** (e.g., retinal imaging, digital cognitive tests).

2. Disease-Modifying Therapies (DMTs). **Target amyloid & tau more effectively** (e.g., next-gen anti-amyloid mAbs, tau vaccines). **Expand beyond plaques/tangles** (e.g., α-synuclein, TDP-43 targeting).

3. Precision Medicine Approaches. **Genetic subtyping** (e.g., APOE4, TREM2 variants) for personalized treatment. **AI-driven patient stratification** for clinical trials.

4. Anti-Tau Therapeutics. **Tau aggregation inhibitors** (e.g., small molecules, antibodies). **Tau clearance strategies** (e.g., autophagy enhancers).

5. Neuroinflammation Modulation. **Microglia-targeted therapies** (e.g., TREM2 agonists, NLRP3 inhibitors). **Anti-inflammatory repurposing** (e.g., SARM1 inhibitors, JAK/STAT modulators).

6. Synaptic Protection & Repair. **Boost neurotrophic factors** (e.g., BDNF mimetics). **Enhance synaptic plasticity** (e.g., glutamate receptor modulators).

7. Vascular Contributions. **Treat small vessel disease** (e.g., blood-brain barrier repair). **Link between hypertension/diabetes and AD**.

8. Combination Therapies. **Amyloid + tau + anti-inflammatory cocktails**. **DMTs + lifestyle interventions** (e.g., exercise, diet synergies).

9. Non-Amyloid Targets. **Mitochondrial dysfunction** (e.g., mitophagy enhancers). **Metabolic reprogramming** (e.g., ketogenic therapy optimization).

10. APOE4-Targeted Therapies. **Gene therapy/editing** (e.g., APOE2 delivery, CRISPR). **APOE4 conformation correctors**.

11. Gut-Brain Axis Interventions. **Probiotics/prebiotics for cognitive protection**. **Role of gut microbiota in neuroinflammation**.

12. Epigenetic Therapies. **HDAC inhibitors/DNA methylation modulators**. **Reverse age-related epigenetic drift**.

13. Cognitive Resilience Mechanisms. **Why some brains resist pathology?** (e.g., "super-agers"). **Enhance cognitive reserve** (e.g., brain stimulation + cognitive training).

14. Advanced Drug Delivery. **BBB-penetrating technologies** (e.g., exosomes, focused ultrasound). **Intranasal delivery for direct CNS access**.

15. Rapid Clinical Trial Designs. **Better surrogate endpoints** (e.g., digital biomarkers). **Adaptive trial platforms** for faster DMT testing.

16. Prevention Trials. **Primary prevention in high-risk cohorts** (e.g., AHEAD 3-45 study). **Lifestyle interventions** (e.g., FINGER 2.0).

17. Sex-Specific Differences. **Impact of menopause, X-chromosome genes**. **Tailored therapies for women** (higher AD prevalence).

18. Sleep & Circadian Rhythm. **Clearance of toxins via glymphatic system**. **Melatonin/theragnostic approaches**.

19. Palliative & Symptomatic Care. **Better agitation/psychosis treatments** (e.g., pimavanserin optimization). **Non-pharmacological symptom management**.

20. Health Disparities & Access. **Barriers to early diagnosis/treatment in underserved groups**. **Cost reduction of DMTs/biomarker testing**.

Gemini 1-20 are as follows:

1. Developing a Cure or Highly Effective Disease-Modifying Therapies for All Stages of AD. Definition: This encompasses research into therapies that can stop, reverse, or significantly slow the neurodegenerative process, not just manage symptoms. This includes exploring novel targets beyond amyloid and tau, such as inflammation, genetic pathways, metabolic dysregulation, and synaptic health. **Importance:** Current treatments offer modest benefits, primarily slowing cognitive decline. A true cure or highly effective disease-modifying therapy, particularly for later stages of the disease, is the paramount goal to alleviate the immense burden on individuals, families, and healthcare systems.

2. Early and Accurate Diagnosis, Especially in Pre-symptomatic and Prodromal Stages. Definition: Developing highly sensitive, specific, non-invasive, and affordable biomarkers (e.g., blood tests, advanced imaging, digital cognitive assessments) that can detect Alzheimer's pathology years before clinical symptoms appear. **Importance:** Interventions are likely to be most effective when administered early, before significant irreversible brain damage occurs. Accurate early diagnosis is crucial for identifying individuals who could benefit from emerging disease-modifying therapies and for recruiting participants into clinical trials.

3. Understanding and Targeting Non-Amyloid/Tau Pathologies and Mechanisms. Definition: Deepening our understanding of other critical contributors to AD pathogenesis beyond amyloid plaques and tau tangles, such as neuroinflammation, glial cell dysfunction (microglia, astrocytes), vascular pathology, mitochondrial dysfunction, metabolic dysregulation, lysosomal dysfunction, synaptic loss, and the role of chronic stress granules. **Importance:** The failure of many amyloid- and tau-centric trials suggests that AD is more complex than initially thought. Addressing these multifaceted pathways is crucial for developing combination therapies and more effective treatments.

4. Personalized Medicine Approaches and Stratification of Patients. Definition: Identifying distinct subtypes of Alzheimer's disease based on genetic profiles, biomarkers, and clinical presentation, and developing personalized treatment strategies tailored to individual patient characteristics for maximum efficacy and minimal side effects. **Importance:** Just like cancer, Alzheimer's is likely not a single disease. Stratifying patients based on their specific disease drivers will improve clinical trial success and guide targeted therapies.

5. Understanding the Role of Genetics and Gene-Environment Interactions. Definition: Identifying new genetic risk factors beyond APOE4, understanding how these genes interact with environmental factors (e.g., diet, exercise, pollution, sleep) to influence disease risk and progression, and developing gene-targeted therapies or preventative strategies. **Importance:** Genetic insights can reveal fundamental disease mechanisms and identify individuals at higher risk for early intervention.

6. Developing Therapies to Restore Synaptic Function and Neuroprotection. Definition: Research focused on preventing synaptic loss, promoting synaptic plasticity, and protecting neurons from degeneration, as synaptic dysfunction is closely correlated with cognitive decline. **Importance:** Ultimately, AD causes cognitive decline by disrupting the communication between brain cells. Protecting and restoring these connections is a direct route to improving cognitive function.

7. Addressing Lifestyle and Modifiable Risk Factors through Intervention Studies. Definition: Rigorous clinical trials to determine the efficacy of lifestyle interventions (e.g., specific diets, exercise regimens, cognitive training, sleep optimization, management of cardiovascular risk factors like hypertension and diabetes) in preventing or delaying AD onset or progression. **Importance:** Modifiable risk factors offer accessible avenues for prevention and could have a significant public health impact, even if they only delay onset.

8. Developing Novel Drug Delivery Methods to the Brain. Definition: Research into innovative ways to deliver therapeutic agents across the blood-brain barrier efficiently and safely, minimizing systemic side effects (e.g., focused ultrasound, intranasal delivery, nanotechnology, gene therapy vectors). **Importance:** Many promising drugs fail because they cannot adequately reach the brain. Overcoming this barrier is critical for drug development.

9. Improving Symptomatic Treatments for Cognitive and Behavioral Symptoms. Definition: Developing more effective and well-tolerated medications and non-pharmacological interventions for managing cognitive symptoms (memory loss, executive dysfunction) and neuropsychiatric symptoms (agitation, depression, psychosis, sleep disturbances) that significantly impact patient and caregiver quality of life. **Importance:** While a cure is the long-term goal, alleviating the daily burden of symptoms is crucial for those currently living with AD.

10. Understanding the Link Between Systemic Health and Brain Health. Definition: Research exploring the intricate connections between peripheral organ systems (e.g., gut microbiome, liver, kidney, cardiovascular system, endocrine system) and brain health in the context of AD, identifying systemic biomarkers and therapeutic targets. **Importance:** AD is increasingly viewed as a systemic disease, not just a brain disorder. Understanding these links opens new avenues for intervention.

11. Addressing Health Disparities in Alzheimer's Disease. Definition: Research to understand and mitigate the disproportionate burden of AD in various racial, ethnic, socioeconomic, and geographic populations, and to ensure equitable access to diagnosis, treatment, and clinical trial participation. **Importance:** Ensuring that all communities benefit from advancements in AD research and care is a critical ethical and public health priority.

12. Leveraging Artificial Intelligence (AI) and Machine Learning for Drug Discovery and Patient Management. Definition: Utilizing AI for tasks such as identifying novel drug targets, accelerating drug discovery, analyzing complex patient data (genomics, imaging, clinical), predicting disease progression, and personalizing treatment plans. **Importance:** AI has the potential to accelerate research, identify patterns otherwise unseen, and revolutionize the efficiency of AD drug development and patient care.

13. Developing and Validating Non-Pharmacological Interventions and Care Models. Definition: Rigorous research on the effectiveness of non-drug approaches (e.g., cognitive stimulation, exercise programs, music therapy, reminiscence therapy, environmental modifications) and optimal care models for improving quality of life, managing behavioral symptoms, and supporting caregivers. **Importance:** Non-pharmacological interventions are essential components of holistic AD care and can significantly improve patient well-being and reduce caregiver burden.

14. Understanding the Role of the Brain's Glymphatic System and Waste Clearance. Definition: Investigating the glymphatic system, the brain's waste clearance system, and how its dysfunction contributes to the accumulation of amyloid, tau, and other toxic proteins in AD, and developing strategies to enhance its function. **Importance:** Impaired waste clearance could be a fundamental problem in AD, offering a novel therapeutic target.

15. Investigating the Interplay Between Sleep and Alzheimer's Pathology. Definition: Research into how sleep disturbances contribute to AD pathology and progression, and how improving sleep quality might impact disease course and cognitive function. **Importance:** Sleep is critical for brain health and waste clearance, and disruptions are common in AD, suggesting a potential feedback loop.

16. Improving Clinical Trial Design and Efficiency. Definition: Developing more innovative and adaptive clinical trial methodologies, including precision trial designs, pragmatic trials, and biomarker-driven trials, to accelerate the testing of new therapies. **Importance:** Efficient clinical trials are essential to bring effective treatments to patients faster.

17. Exploring Repurposing of Existing Drugs. Definition: Systematic screening and clinical testing of drugs already approved for other conditions to see if they can be repurposed for AD, potentially accelerating therapeutic development due to known safety profiles. **Importance:** Repurposing can be a faster and less costly path to new treatments.

18. Understanding the Role of the Blood-Brain Barrier Dysfunction. Definition: Research into how the integrity and function of the blood-brain barrier are compromised in AD, contributing to pathology and limiting drug delivery, and developing strategies to restore its function. **Importance:** A compromised blood-brain barrier is implicated in disease progression and impacts drug efficacy.

19. Developing Advanced Neuroimaging Techniques for Monitoring Disease Progression and Treatment Response. Definition: Further refinement of imaging technologies (e.g., PET, MRI) to provide more detailed,

sensitive, and quantitative measures of pathology (amyloid, tau, inflammation, synaptic density) and neuronal function, allowing better monitoring of disease and treatment effects. **Importance:** Advanced imaging is critical for understanding disease mechanisms, early diagnosis, and assessing the effectiveness of new therapies in clinical trials.

20. Enhancing Data Sharing, Collaboration, and Open Science Initiatives. Definition: Fostering greater collaboration among researchers globally and establishing platforms for widespread sharing of research data, biological samples, and clinical trial results to accelerate discovery. **Importance:** Open science and data sharing prevent duplication of effort, maximize the utility of valuable research resources, and speed up the pace of discovery.

Perplexity 1-20 are as follows:

1. Disease-Modifying Therapies. Develop treatments that slow, halt, or reverse the underlying disease process, not just manage symptoms.

2. Early and Accurate Diagnosis. Advance biomarkers, imaging, and other diagnostic tools to detect Alzheimer's at the earliest, most treatable stages.

3. Understanding Disease Mechanisms. Deepen knowledge of the biological, genetic, and environmental factors that drive Alzheimer's and related dementias.

4. Prevention Strategies. Identify and test interventions to reduce risk or delay onset, including lifestyle, pharmacological, and public health approaches.

5. Addressing Drug Resistance and Treatment Failure. Investigate why some patients do not respond to current treatments and develop strategies to overcome these barriers.

6. Personalized and Precision Medicine. Tailor treatments based on individual genetic, molecular, and clinical profiles for more effective and less toxic therapies.

7. Enhancing Symptom Management. Improve therapies for cognitive, behavioral, and psychological symptoms to enhance quality of life for patients and families.

8. Support for Caregivers and Families. Develop and evaluate interventions that support caregivers' health, wellbeing, and ability to provide care.

9. Improving Quality and Delivery of Care. Optimize care models across settings (home, hospital, long-term care) and ensure evidence-based practices are implemented.

10. Reducing Health Disparities. Address inequalities in diagnosis, treatment, and outcomes across diverse populations and communities.

11. Clinical Trial Innovation and Access. Design more efficient, inclusive, and adaptive clinical trials to accelerate therapeutic development and patient access.

12. Non-Pharmacological Interventions. Research the effectiveness of lifestyle, cognitive training, and psychosocial therapies in delaying progression and improving outcomes.

13. Integration of Digital Health and AI. Leverage digital tools, artificial intelligence, and telemedicine to improve diagnosis, monitoring, and personalized care.

14. Understanding and Managing Comorbidities. Study and address the impact of other health conditions (e.g., cardiovascular disease, diabetes) on Alzheimer's progression and treatment.

15. Advancing Biomarker Discovery. Identify new blood, imaging, and genetic biomarkers to track disease progression and treatment response.

16. Enhancing Long-Term Outcomes and Survivorship. Research long-term effects of Alzheimer's and its treatments, including aging, quality of life, and chronic disease management.

17. Palliative and End-of-Life Care. Improve approaches to palliative care and support for advanced-stage patients and their families.

18. Workforce Training and Professional Development. Develop training programs to equip healthcare professionals with the skills needed for high-quality dementia care.

19. Socioeconomic and Policy Research. Study the socioeconomic impact of Alzheimer's and evaluate public health policies to inform effective resource allocation.

20. Community Engagement and Public Awareness. Promote awareness, reduce stigma, and increase community involvement in research and care initiatives.

A2: Cancer

ChatGPT 1-20 are as follows:

1. Understanding and Targeting Cancer Heterogeneity. Goal: Address tumor diversity within and across patients. **Why it matters**: Intra- and inter-tumor heterogeneity leads to treatment resistance and recurrence. **Example**: Single-cell genomics to map mutations and cell types.

2. Overcoming Drug Resistance Mechanisms. Goal: Prevent or reverse resistance to therapies. **Why it matters**: Resistance limits long-term success of targeted therapies and chemotherapy. **Example**: Studying adaptive resistance pathways (e.g., PI3K, KRAS, p53).

3. Enhancing Immunotherapy Efficacy. Goal: Expand durable responses to checkpoint inhibitors and CAR-T cells. **Why it matters**: Many patients don't respond to immunotherapy or relapse. **Example**: Targeting T-cell exhaustion and tumor microenvironment (TME) suppression.

4. Early Detection and Interception of Cancer. Goal: Detect cancer at pre-malignant or early stages. **Why it matters**: Survival is much higher when cancer is caught early. **Example**: Liquid biopsies using ctDNA, exosomes, or methylation markers.

5. Tumor Microenvironment Modulation. Goal: Alter stromal, immune, and metabolic support systems of tumors. **Why it matters**: The TME promotes growth, evades immune response, and affects drug delivery. **Example**: Targeting cancer-associated fibroblasts or hypoxia.

6. Personalized and Precision Oncology. Goal: Match therapies to individual tumor and patient profiles. **Why it matters**: Tailored therapies improve outcomes and reduce toxicity. **Example**: Genomic and transcriptomic profiling for clinical decision-making.

7. Developing Novel Therapeutic Modalities. Goal: Create new drug classes beyond current chemotherapies and biologics. **Why it matters**: Many cancers lack effective treatments. **Example**: Protein degraders (PROTACs), RNA-based therapies, bispecific antibodies.

8. Cancer Prevention and Risk Stratification. Goal: Reduce incidence via risk-based screening and interventions. **Why it matters**: Prevention can have greater population-level impact than treatment. **Example**: Genetic testing for BRCA, Lynch syndrome; lifestyle and microbiome modulation.

9. Minimal Residual Disease (MRD) Monitoring. Goal: Detect microscopic disease post-treatment to predict relapse. **Why it matters**: MRD detection enables early intervention and adaptive therapy. **Example**: NGS-based detection of ctDNA after surgery or chemotherapy.

10. Pediatric and Rare Cancers. Goal: Improve outcomes where research is sparse. **Why it matters**: Many rare and pediatric cancers lack clinical trials and targeted therapies. **Example**: Epigenetic therapies for pediatric gliomas or sarcomas.

11. Cancer Metabolism and Stress Adaptation. Goal: Target metabolic dependencies and stress responses of tumors. **Why it matters**: Metabolic plasticity contributes to growth and survival under therapy. **Example**: Inhibiting glutamine metabolism or autophagy pathways.

12. Combination Therapy Optimization. Goal: Rationally design drug combinations that enhance efficacy and reduce toxicity. **Why it matters**: Synergistic treatments can prevent resistance and relapse. **Example**: Timing of immunotherapy with radiotherapy or anti-angiogenics.

13. AI and Computational Oncology. Goal: Use machine learning to predict progression, stratify patients, and design trials. **Why it matters**: Data-driven decisions can optimize care and reduce trial failures. **Example**: AI-guided drug repurposing or image-based diagnostics.

14. Real-World Evidence and Adaptive Clinical Trials. Goal: Make trials faster, more inclusive, and responsive to real-world patients. **Why it matters**: Traditional trials are slow and often exclude complex patients. **Example**: Basket trials, umbrella trials, and platform trials.

15. Microbiome-Cancer Interactions. Goal: Understand and manipulate gut and tumor microbiomes to affect outcomes. **Why it matters**: The microbiome can modulate immune response and drug metabolism. **Example**: Fecal microbiota transplant (FMT) to enhance immunotherapy.

16. Epigenetic Reprogramming. Goal: Reverse aberrant gene expression without altering DNA. **Why it matters**: Epigenetic dysregulation drives many aggressive cancers. **Example**: BET inhibitors or HDAC inhibitors for resistant tumors.

17. Improving Cancer Survivorship and QoL. Goal: Minimize long-term effects of cancer and its treatment. **Why it matters**: Millions live with chronic effects like fatigue, pain, or infertility. **Example**: Rehabilitation, cardio-oncology, cognitive support, fertility preservation.

18. Cancer Stem Cells and Cellular Hierarchies. Goal: Eradicate self-renewing cancer cell populations responsible for relapse. **Why it matters**: CSCs are often resistant to standard therapy. **Example**: Inhibiting Wnt, Notch, or Hedgehog pathways.

19. Addressing Disparities in Cancer Outcomes. Goal: Ensure equitable access to screening, diagnosis, and treatment. **Why it matters**: Race, geography, and socioeconomic status affect survival. **Example**: Community-based interventions, telehealth, policy reforms.

20. Enhancing Drug Delivery Systems. Goal: Improve precision and efficiency of drug targeting to tumors. **Why it matters**: Poor delivery reduces efficacy and increases side effects. **Example**: Nanoparticles, antibody-drug conjugates (ADCs), or local depot systems.

Claude 1-20 are as follows:

1. Personalized Medicine and Biomarker Development Developing comprehensive biomarker panels to predict treatment response and resistance patterns for individual patients. This enables precise treatment selection and monitoring, potentially transforming outcomes across all cancer types.

2. Immunotherapy Enhancement and Combination Strategies Advancing immune checkpoint inhibitors, CAR-T cell therapy, and novel immunomodulatory approaches. Research into combination therapies and methods to overcome immune resistance could benefit the majority of cancer patients.

3. Early Detection and Screening Technologies Creating highly sensitive, specific, and cost-effective screening methods including liquid biopsies, AI-powered imaging, and multi-cancer early detection assays. Early detection remains the most impactful factor in cancer survival.

4. Tumor Microenvironment Modulation Understanding and targeting the complex ecosystem surrounding tumors, including stromal cells, blood vessels, and immune infiltrates. This approach could enhance the effectiveness of existing treatments.

5. Metastasis Prevention and Treatment Developing therapies specifically targeting the metastatic cascade, as metastatic disease remains the primary cause of cancer death. Research into circulating tumor cells and dormant metastases is crucial.

6. Drug Resistance Mechanisms Investigating molecular mechanisms of both intrinsic and acquired resistance to targeted therapies, chemotherapy, and immunotherapy. Understanding resistance could lead to more durable treatments.

7. Cancer Stem Cell Biology Researching cancer stem cells and their role in treatment resistance, recurrence, and metastasis. Targeting these cells could prevent relapse and improve long-term survival.

8. Artificial Intelligence in Cancer Care Developing AI systems for diagnosis, treatment planning, drug discovery, and outcome prediction. AI could accelerate research and improve clinical decision-making across all aspects of cancer care.

9. Liquid Biopsy Advancement Improving circulating tumor DNA, RNA, and protein detection for monitoring treatment response, detecting minimal residual disease, and surveillance. This non-invasive approach could revolutionize cancer monitoring.

10. Combination Therapy Optimization Systematically studying optimal combinations of existing and new treatments, including sequencing, dosing, and timing. Most future cancer treatments will likely involve combinations.

11. Pediatric Cancer Research Developing age-appropriate treatments for childhood cancers, which often have different biology than adult cancers. This includes addressing long-term effects of treatment in survivors.

12. Rare Cancer Research Investigating orphan cancers and developing targeted therapies for rare malignancies. While individually uncommon, rare cancers collectively affect many patients and are often neglected.

13. Epigenetic Therapy Development Advancing epigenetic modulators and understanding their role in cancer development and treatment. Epigenetic changes are reversible, offering unique therapeutic opportunities.

14. Therapeutic Delivery Systems Developing novel drug delivery methods including nanoparticles, antibody-drug conjugates, and targeted delivery systems to improve efficacy while reducing toxicity.

15. Tumor Heterogeneity Research Understanding spatial and temporal tumor heterogeneity and its implications for treatment. This knowledge could inform adaptive treatment strategies.

16. Supportive Care and Quality of Life Researching methods to prevent and manage treatment-related side effects, improve symptom control, and enhance patient quality of life throughout the cancer journey.

17. Cancer Prevention Strategies Developing and validating prevention approaches including vaccines, chemoprevention, and lifestyle interventions. Prevention remains more effective than treatment.

18. Precision Surgery and Radiation Advancing minimally invasive surgical techniques, image-guided surgery, and precision radiation therapy to improve outcomes while reducing morbidity.

19. Survivorship Research Investigating long-term effects of cancer treatment, developing survivorship care plans, and addressing late effects. As more patients survive cancer, this becomes increasingly important.

20. Global Cancer Care Disparities Researching and addressing disparities in cancer care access, outcomes, and treatment effectiveness across different populations and geographic regions.

DeepSeek 1-20 are as follows:

1. Early Detection & Biomarkers. Develop non-invasive, highly sensitive tests (e.g., liquid biopsies, multi-cancer early detection). Identify biomarkers for pre-malignant conditions.

2. Immunotherapy Advancements. Overcome resistance to checkpoint inhibitors (PD-1/CTLA-4). Expand CAR-T cell efficacy to solid tumors.

3. Precision Oncology & Targeted Therapies. Improve genomic and proteomic profiling for personalized treatment. Develop drugs against "undruggable" targets (e.g., KRAS, MYC).

4. Tumor Microenvironment (TME) Manipulation. Disrupt cancer-stromal interactions (e.g., TGF-β, hypoxia). Enhance immune infiltration in "cold" tumors.

5. Metastasis Prevention & Treatment. Understand and block metastatic seeding mechanisms. Develop therapies targeting dormant disseminated cells.

6. AI & Computational Oncology. AI-driven drug discovery and treatment optimization. Predictive modeling for resistance and recurrence.

7. Epigenetic Therapies. Develop drugs targeting DNA methylation/histone modifiers. Reverse therapy-induced epigenetic resistance.

8. Cancer Metabolism Interventions. Exploit metabolic vulnerabilities (e.g., glutamine dependence). Combine metabolic inhibitors with immunotherapy.

9. Microbiome & Cancer Immunity. Define gut microbiome's role in treatment response. Develop microbiome-modulating therapies.

10. Resistance Mechanisms. Overcome intrinsic and acquired drug resistance. Develop adaptive therapy strategies.

11. Pediatric & Rare Cancers. Identify novel targets in understudied cancers. Develop less toxic therapies for children.

12. Radiotherapy Enhancements. Improve tumor-targeted radiation (e.g., FLASH, proton therapy). Combine radiotherapy with immunomodulators.

13. Cancer Vaccines. Develop preventive vaccines (e.g., HPV-like for other cancers). Therapeutic mRNA vaccines for personalized neoantigens.

14. Senescence & Aging in Cancer. Understand how aging promotes tumorigenesis. Exploit senolytic therapies to prevent recurrence.

15. Combination Therapy Optimization. Define best drug sequences and synergies. Reduce overlapping toxicities.

16. Liquid Biopsy & MRD Detection. Improve sensitivity for minimal residual disease (MRD). Use circulating tumor DNA (ctDNA) for real-time monitoring.

17. Cancer Neuroscience. Study neural signaling in tumor growth. Block perineural invasion in aggressive cancers.

18. Health Disparities & Access. Ensure equitable global access to breakthroughs. Study biological vs. socioeconomic drivers of disparities.

19. Supportive & Survivorship Care. Reduce long-term side effects (e.g., cardiotoxicity, neuropathy). Improve quality of life during and after treatment.

20. Preventive Oncology. Develop better chemopreventive agents. Study lifestyle interventions (diet, exercise) in high-risk groups.

Gemini 1-20 are as follows:

1. Developing Personalized/Precision Oncology Approaches. Definition: Tailoring treatments to an individual's specific tumor characteristics, including genetic mutations, protein expression, and tumor microenvironment, using technologies like genomic sequencing and liquid biopsies. **Importance:** This is paramount. Cancers are highly heterogeneous, and a "one-size-fits-all" approach often leads to resistance and suboptimal outcomes. Precision oncology aims to maximize efficacy and minimize side effects by targeting the specific vulnerabilities of each patient's cancer. This includes the development of personalized cancer vaccines.

2. Enhancing Immunotherapy Efficacy and Overcoming Resistance. Definition: Research into improving existing immunotherapies (e.g., checkpoint inhibitors, CAR T-cell therapy) and developing new ones, while also understanding and overcoming mechanisms of resistance that limit their effectiveness in many patients. **Importance:** Immunotherapy has revolutionized cancer treatment, but it only works for a subset of patients and resistance is a major challenge. Unlocking broader and more durable responses is critical.

3. Early Detection and Diagnosis Technologies. Definition: Developing highly sensitive and specific methods for detecting cancer at its earliest stages, ideally before symptoms appear, through non-invasive techniques like blood tests (liquid biopsies), advanced imaging, and AI-powered analysis of health data. **Importance:** Early diagnosis is consistently linked to better treatment outcomes and higher survival rates across almost all cancer types.

4. Understanding and Targeting Cancer Metastasis. Definition: Investigating the complex biological processes by which cancer cells spread from the primary tumor to distant sites, and developing treatments that specifically inhibit or reverse this metastatic process. **Importance:** Metastasis is responsible for the vast majority of cancer-related deaths. Effective treatments for metastatic disease are urgently needed.

5. Minimizing and Managing Treatment-Related Side Effects and Long-Term Toxicities. Definition: Research into strategies to reduce the immediate and long-term adverse effects of cancer treatments (chemotherapy, radiation, targeted therapies, immunotherapies) on patients' quality of life, including fatigue, pain, neuropathy, and organ damage. This also includes understanding the biological basis of these side effects. **Importance:** While survival is paramount, the burden of treatment can be immense. Improving patient experience and long-term health is a key priority.

6. Developing Novel Drug Delivery Systems. Definition: Research into innovative ways to deliver therapeutic agents directly and efficiently to tumor cells, minimizing systemic toxicity. This includes nanoparticles, targeted conjugates, and localized delivery methods (e.g., magnetic drug delivery for brain tumors). **Importance:** Efficient and targeted drug delivery can significantly enhance treatment efficacy and reduce side effects.

7. Overcoming Drug Resistance Mechanisms. Definition: Identifying the molecular mechanisms by which cancer cells develop resistance to various therapies (chemotherapy, targeted drugs, immunotherapy) and designing new strategies or combination therapies to circumvent these resistances. **Importance:** Drug resistance is a major cause of treatment failure and relapse.

8. Improving Cancer Prevention Strategies. Definition: Research into identifying and mitigating cancer risk factors (lifestyle, environmental, genetic), developing effective preventative vaccines, and implementing population-level interventions. **Importance:** Preventing cancer from developing in the first place is the ultimate goal, reducing the need for treatment altogether.

9. Integrating Artificial Intelligence (AI) and Machine Learning in Cancer Care. Definition: Utilizing AI for tasks such as drug discovery, biomarker identification, treatment planning, image analysis for diagnosis, predicting treatment response, and personalizing care pathways. **Importance:** AI has the potential to accelerate discovery, optimize treatment decisions, and improve efficiency across the entire cancer care continuum.

10. Understanding the Tumor Microenvironment (TME). Definition: Research into the complex interactions between cancer cells and their surrounding non-cancerous cells, blood vessels, and immune cells within the tumor, and how this environment influences tumor growth, metastasis, and response to therapy. **Importance:** The TME plays a critical role in cancer progression and resistance, offering new targets for treatment.

11. Optimizing Combination Therapies. Definition: Investigating the most effective combinations of different treatment modalities (e.g., immunotherapy + chemotherapy + radiation + targeted therapy) to achieve synergistic effects and improve patient outcomes. **Importance:** Combining treatments often yields better results than monotherapy, but optimizing these combinations is complex.

12. Improving Supportive and Palliative Care. Definition: Research focused on enhancing the quality of life for cancer patients throughout their journey, including symptom management, psychological support, and end-of-life care. **Importance:** Beyond direct tumor treatment, addressing the holistic needs of patients and their families is crucial.

13. Addressing Health Inequalities in Cancer Outcomes. Definition: Research into the socioeconomic, ethnic, geographic, and other factors that contribute to disparities in cancer incidence, diagnosis, treatment access, and outcomes, and developing interventions to reduce these inequalities. **Importance:** Ensuring equitable access to effective care for all populations is a fundamental ethical and public health priority.

14. Advanced Imaging Techniques. Definition: Developing new and improved imaging technologies for more precise tumor detection, characterization, treatment response monitoring, and guidance during surgery or radiation therapy. **Importance:** Better imaging leads to more accurate diagnosis, staging, and treatment assessment.

15. Investigating the Role of the Microbiome in Cancer. Definition: Researching how the collection of microorganisms in and on the body influences cancer development, progression, and response to therapies, particularly immunotherapy. **Importance:** The microbiome is an emerging area with potential for novel therapeutic interventions.

16. Developing Cancer Vaccines (Prophylactic and Therapeutic). Definition: Continued research and development of vaccines to prevent certain cancers (e.g., HPV vaccine) and therapeutic vaccines designed to stimulate the immune system to attack existing cancer cells. **Importance:** Vaccines offer a powerful approach for prevention and long-term control.

17. Understanding Cancer Cachexia and Metabolic Reprogramming. Definition: Research into the severe wasting syndrome (cachexia) often experienced by cancer patients, and the metabolic changes within cancer cells that fuel their growth, with the aim of developing interventions to counter these processes. **Importance:** Cachexia significantly impacts quality of life and treatment tolerance. Targeting cancer metabolism offers new therapeutic avenues.

18. Improving Surgical Techniques and Technologies. Definition: Research into less invasive surgical procedures, image-guided surgery, robotic surgery, and techniques to ensure complete tumor removal while preserving healthy tissue. **Importance:** Surgery remains a cornerstone of treatment for many solid tumors, and advancements can improve outcomes and reduce morbidity.

19. Enhancing Radiotherapy Precision and Efficacy. Definition: Developing advanced radiotherapy techniques (e.g., proton therapy, stereotactic body radiation therapy, adaptive radiotherapy) that deliver highly targeted radiation doses with minimal damage to surrounding healthy tissues, and combining radiation with other therapies. **Importance:** Radiation therapy is vital, and increased precision can lead to better local control and fewer side effects.

20. Establishing Robust Data Infrastructure and Biobanks. Definition: Creating comprehensive and interoperable databases of patient data (genomic, clinical, treatment response) and high-quality biobanks of tumor and blood samples to facilitate large-scale research and enable discovery. **Importance:** Access to high-quality, standardized data and biological samples is fundamental for all areas of cancer research, enabling discovery and validation of new insights.

Perplexity 1-20 are as follows:

1. Early Detection and Screening. Developing sensitive, specific, and affordable methods for detecting cancer at its earliest stages, including blood-based biomarkers and advanced imaging.

2. Understanding and Overcoming Drug Resistance. Investigating mechanisms of resistance to current therapies and designing strategies to prevent or reverse resistance.

3. Precision Medicine and Genomics. Tailoring treatments to individual genetic, molecular, and cellular profiles for more effective and less toxic therapies.

4. Immunotherapy Optimization. Advancing immune-based treatments, including checkpoint inhibitors, CAR-T cells, and cancer vaccines, and identifying biomarkers for response.

5. Targeting Metastasis. Understanding and preventing the spread of cancer, which is the leading cause of cancer-related deaths.

6. Tumor Microenvironment Research. Studying the tumor's surrounding environment to develop therapies that disrupt cancer-supporting conditions.

7. Cancer Prevention Strategies. Identifying risk factors, developing vaccines, and implementing lifestyle interventions to reduce cancer incidence.

8. Novel Therapeutic Modalities. Discovering and testing new classes of drugs, such as antibody-drug conjugates, RNA-based therapies, and targeted protein degraders.

9. Cancer Biology and Mechanisms of Formation. Investigating the fundamental processes that drive cancer initiation and progression.

10. Liquid Biopsies and Non-Invasive Diagnostics. Developing blood and stool tests for early detection, monitoring, and recurrence surveillance.

11. Addressing Cancer Disparities. Researching and reducing inequalities in cancer outcomes related to race, ethnicity, geography, and socioeconomic status.

12. Artificial Intelligence and Computational Biology. Applying AI and machine learning to improve diagnostics, treatment planning, and drug discovery.

13. Improving Radiotherapy and Imaging. Enhancing the effectiveness and precision of radiotherapy, and developing advanced imaging to guide treatment.

14. Microbiome and Cancer. Exploring how the gut and tumor microbiome influence cancer risk, progression, and treatment response.

15. Organoid and Model Systems. Using patient-derived organoids and advanced models to personalize therapy and predict drug response.

16. Overcoming Treatment Side Effects and Toxicities. Researching ways to minimize and manage acute and long-term side effects of cancer therapies.

17. Circulating Tumor Cells and Minimal Residual Disease. Tracking cancer cells in the bloodstream to predict relapse and guide therapy.

18. Palliative and Supportive Care. Improving quality of life for patients by addressing pain, psychological, and social needs during and after treatment.

19. Clinical Trial Innovation and Access. Designing more efficient, inclusive, and adaptive clinical trials to accelerate therapeutic development and patient access.

20. Survivorship and Long-Term Outcomes. Studying the long-term effects of cancer and its treatment to optimize survivorship care and monitor late effects.

A3: COVID-19

ChatGPT 1-20 are as follows:

1. Development of Broad-Spectrum Antiviral Therapies. Goal: Discover drugs that target current and future SARS-CoV-2 variants. **Why it matters**: SARS-CoV-2 continues to evolve; pan-coronavirus antivirals would future-proof treatment. **Example**: Inhibitors of viral proteases, polymerases, or entry mechanisms (e.g., 3CLpro, RdRp).

2. Understanding and Treating Long COVID. Goal: Identify causes and treatments for persistent post-infection symptoms. **Why it matters**: Long COVID affects millions globally with disabling symptoms. **Example**: Trials of antivirals, anti-inflammatories, and mitochondrial or autonomic modulators.

3. Development of Variant-Resistant Monoclonal Antibodies. Goal: Create antibodies that retain potency across variants. **Why it matters**: Most previous monoclonal therapies have lost efficacy due to viral mutations. **Example**: Targeting conserved viral epitopes with bispecific antibodies or antibody cocktails.

4. Host-Directed Therapies. Goal: Target host pathways that support viral replication or drive severe disease. **Why it matters**: Less susceptible to viral resistance and may benefit broad populations. **Example**: TMPRSS2 inhibitors, JAK inhibitors, statins, or immune-modulating drugs.

5. Early Outpatient Treatment Strategies. Goal: Prevent progression to severe disease using interventions at symptom onset. **Why it matters**: Early treatment can dramatically reduce hospitalization and death. **Example**: Paxlovid timing optimization, combination antiviral trials, inhaled therapies.

6. Immune Response Modulation in Severe Disease. Goal: Fine-tune inflammatory and immune responses to avoid cytokine storm or immune suppression. **Why it matters**: Immunopathology, not viral load, often drives critical illness. **Example**: Corticosteroids, IL-6 inhibitors (tocilizumab), IL-1 blockers, or novel immune modulators.

7. Optimizing Treatment for High-Risk Populations. Goal: Customize therapies for elderly, immunocompromised, or comorbid patients. **Why it matters**: These groups experience worse outcomes and may respond differently to drugs. **Example**: Alternative dosing, passive immunization, or pre-exposure prophylaxis.

8. Combination Antiviral Therapies. Goal: Use drug combinations to prevent resistance and improve efficacy. **Why it matters**: Single-drug regimens (e.g., Paxlovid alone) may lead to viral rebound or resistance. **Example**: Paxlovid + molnupiravir trials, or combining direct-acting antivirals with host-targeted agents.

9. Understanding Mechanisms of Viral Persistence and Rebound. Goal: Explain why some patients relapse after treatment or show persistent virus. **Why it matters**: Crucial to improving therapy duration and preventing transmission. **Example**: Tissue reservoir studies, immunologic profiling of rebound cases.

10. Treatment of Post-Acute Sequelae in COVID-19 Survivors. Goal: Address organ damage and dysfunction from severe infection. **Why it matters**: Cardiovascular, pulmonary, renal, and neurologic complications persist post-infection. **Example**: Antifibrotic drugs, neuroprotective agents, rehabilitation strategies.

11. Personalized and Precision COVID-19 Treatment. Goal: Stratify patients based on biomarkers, genomics, or immune profiles to guide treatment. **Why it matters**: Reduces overtreatment and improves outcomes. **Example**: Biomarker-guided corticosteroid use, cytokine profiling.

12. Targeting Viral Entry and Fusion Mechanisms. Goal: Inhibit the virus's ability to enter human cells. **Why it matters**: Blocks infection at its earliest stage. **Example**: ACE2 decoys, fusion inhibitors, entry receptor blockade.

13. Repurposing Existing Drugs with Antiviral or Immunomodulatory Effects. Goal: Leverage known safety profiles to rapidly identify new treatments. **Why it matters**: Faster and cheaper than novel drug development. **Example**: Metformin, fluvoxamine, statins, antihistamines.

14. Addressing Viral Evolution and Resistance to Treatment. Goal: Monitor and counteract antiviral resistance in real time. **Why it matters**: Resistance can render treatments ineffective across large populations. **Example**: Global surveillance networks, real-time genomic resistance detection.

15. Strategies for Preventing Nosocomial (Hospital-Acquired) COVID-19. Goal: Prevent transmission and reinfection in healthcare settings. **Why it matters**: Hospitals amplify severe disease spread, especially to high-risk patients. **Example**: Environmental decontamination, prophylactic antivirals for staff/patients.

16. Treatment Optimization for Pediatric Populations. Goal: Ensure safety and efficacy in children, including those with MIS-C. **Why it matters**: Pediatric immune responses and pharmacokinetics differ from adults. **Example**: Pediatric trials of antivirals and immunomodulators.

17. COVID-19 and Co-Infections. Goal: Improve outcomes in patients co-infected with influenza, RSV, or bacterial pathogens. **Why it matters**: Co-infections worsen prognosis and complicate management. **Example**: Combined antiviral-antibiotic protocols; rapid multiplex diagnostics.

18. Affordable Treatment Access in Low-Resource Settings. Goal: Ensure global equity in accessing effective COVID-19 therapies. **Why it matters**: Disparities in access worsen global disease burden and variant evolution. **Example**: Patent waivers, generic manufacturing, decentralized treatment trials.

19. AI-Driven Drug Discovery and Treatment Optimization. Goal: Use machine learning to identify novel compounds or predict optimal therapies. **Why it matters**: Speeds up drug repurposing and personalized treatment. **Example**: Predictive models for cytokine response, AI-based trial design.

20. Integrating COVID-19 Treatment with Broader Respiratory Disease Platforms. Goal: Manage COVID-19 as part of integrated respiratory illness care. **Why it matters**: Sustainable systems are needed as COVID-19 becomes endemic. **Example**: Co-treatment guidelines, combined clinical pathways for COVID/influenza/RSV.

Claude 1-20 are as follows:

1. Long COVID Prevention and Treatment Despite ongoing research efforts, there is still no specific treatment for long COVID, which affects millions worldwide. Research into biomarkers, targeted therapies, and prevention strategies is critical as this represents the most pressing unmet medical need.

2. Next-Generation Antiviral Development Developing more effective antivirals with broader activity against SARS-CoV-2 variants, improved oral bioavailability, and reduced drug interactions. Efficacy of antivirals can be improved by combining different therapeutic classes while preserving economical sustainability.

3. Variant-Resistant Therapeutic Strategies Creating treatments that maintain effectiveness against emerging variants, as global SARS-CoV-2 variant trends have slightly shifted with new variants like NB.1.8.1 increasing globally.

4. Improved Oral Antiviral Formulations Enhancing existing medications like Paxlovid and developing new oral antivirals with better tolerability, fewer drug interactions, and broader therapeutic windows for home-based treatment.

5. Post-Acute Sequelae Biomarker Development Biomarkers like IL-6 and genetic factors may soon help identify at-risk individuals early, enabling preventive strategies for long COVID prevention and management.

6. Respiratory Delivery Systems Respiratory delivery should be better investigated in clinical trials to improve drug targeting to the primary site of infection and reduce systemic side effects.

7. Immune System Modulation Developing therapies that address persistent immune dysfunction and inflammation associated with both acute COVID-19 and long COVID, including targeted immunomodulatory agents.

8. Combination Therapy Optimization Researching optimal combinations of antivirals, immunomodulators, and supportive therapies to maximize efficacy while minimizing resistance development and adverse effects.

9. Pediatric Treatment Development Creating age-appropriate formulations and dosing strategies for children, addressing the unique challenges of COVID-19 in pediatric populations.

10. Preventive Therapies for High-Risk Groups For the immunocompromised people, pre-exposure prophylaxis with Pemgarda can help reduce the acute, severe symptomatic infections, but broader preventive strategies are needed.

11. Neurological Manifestation Treatment Developing targeted therapies for COVID-19-associated neurological complications, including brain fog, cognitive dysfunction, and other central nervous system effects.

12. Personalized Treatment Approaches Using genetic, immunological, and clinical markers to tailor treatment selection and dosing for individual patients, improving outcomes and reducing adverse effects.

13. Viral Persistence Research Understanding mechanisms of viral persistence in various tissue compartments and developing strategies to clear persistent viral reservoirs that may contribute to long COVID.

14. Inflammatory Response Modulation Researching targeted anti-inflammatory therapies that can prevent or treat the hyperinflammatory responses associated with severe COVID-19 and long-term complications.

15. Coinfection and Comorbidity Management Developing treatment strategies for patients with COVID-19 and concurrent infections or chronic conditions, optimizing outcomes in complex clinical scenarios.

16. Rapid Diagnostic-Therapeutic Integration Creating point-of-care testing systems that can quickly identify viral variants and resistance patterns to guide immediate treatment decisions.

17. Global Access and Equity Research Continuing to work toward providing equitable access is essential to enable all populations to receive preventive care and treatments, particularly in resource-limited settings.

18. Treatment Resistance Monitoring Developing surveillance systems to detect emerging resistance to antivirals and other therapeutics, enabling rapid response to maintain treatment effectiveness.

19. Mental Health and Psychological Support Researching integrated approaches to address the psychological impacts of COVID-19, including anxiety, depression, and post-traumatic stress associated with the illness.

20. Healthcare System Optimization Studying optimal care delivery models, telemedicine integration, and healthcare system preparedness for managing COVID-19 treatment at population scale.

DeepSeek 1-20 are as follows:

1. Broad-Spectrum Antivirals. Develop pan-coronavirus drugs (e.g., targeting conserved viral proteases or polymerases). Improve oral antivirals (e.g., next-gen Paxlovid with fewer drug interactions).

2. Long COVID Mechanisms & Treatments. Define pathophysiology (viral persistence, autoimmunity, endothelial dysfunction). Test targeted therapies (e.g., antivirals, immunomodulators, mitochondrial support).

3. Next-Gen Vaccines. Universal coronavirus vaccines (e.g., nanoparticle/mRNA designs against spike variants). Mucosal vaccines to block transmission (intranasal/spray formulations).

4. Immune Modulation for Severe Cases. Optimize timing/targets for immunomodulators (e.g., IL-6/JAK inhibitors, complement blockers). Prevent cytokine storms without immunosuppression.

5. Early Biomarkers for Disease Progression. Predict severe disease using multi-omics (e.g., proteomics, transcriptomics). Develop point-of-care tests to guide treatment escalation.

6. Monoclonal Antibodies (mAbs) for Emerging Variants. Engineer variant-proof mAbs (e.g., bispecific antibodies). Improve delivery (e.g., inhaled mAbs for early infection).

7. Antiviral Resistance Prevention. Monitor resistance mutations to remdesivir, nirmatrelvir, etc. Develop high-barrier-to-resistance drugs.

8. Pediatric & Immunocompromised Treatments. Tailor antivirals/immunotherapies for high-risk children. Prevent persistent infection in immunocompromised hosts.

9. Repurposed Drugs with Rigorous Evidence. Validate promising candidates (e.g., fluvoxamine, bromhexine) in large trials. Clarify roles of anticoagulants/statins in acute/post-COVID care.

10. Combination Therapies. Synergistic drug pairs (e.g., antiviral + immunomodulator). Sequential protocols for different disease phases.

11. Acute Respiratory Support Innovations. Non-invasive oxygenation strategies to avoid intubation. Anti-fibrotics to prevent post-COVID lung damage.

12. Neurological & Cognitive Protection. Prevent/treat neuro-COVID (e.g., microclot inhibitors, NMDA antagonists). Address anosmia, brain fog, and neuroinflammation.

13. Rapid, Affordable Global Diagnostics. Low-cost PCR alternatives for resource-limited settings. Home tests differentiating COVID from flu/RSV.

14. Post-Exposure Prophylaxis. Develop early-interception drugs (e.g., nasal sprays, fast-acting mAbs).

15. Epigenetic & Metabolic Therapies. Target COVID-induced epigenetic dysregulation (e.g., HDAC inhibitors). Correct mitochondrial dysfunction in long COVID.

16. AI-Driven Personalized Treatment. Machine learning to predict optimal drug regimens. Real-world data analytics for emerging variant responses.

17. Structural Biology for Drug Design. Cryo-EM/molecular dynamics to identify new viral vulnerabilities.

18. Global Equity in Treatment Access. Scale up antiviral production/distribution in low-income countries. Overcome cold-chain barriers for biologics.

19. One Health & Zoonotic Surveillance. Monitor animal reservoirs to preempt spillover variants.

20. Behavioral & Public Health Strategies. Improve treatment adherence (e.g., shorter courses, better-tasting pediatrics). Combat misinformation driving vaccine/therapy hesitancy.

Gemini 1-20 are as follows:

1. Understanding and Treating Post-Acute Sequelae of COVID-19 (PASC) / Long COVID. Definition: Research into the underlying mechanisms (e.g., viral persistence, autoimmunity, microclotting, immune dysregulation) of chronic symptoms experienced by individuals after acute COVID-19 infection, and the development of effective diagnostic tools and targeted treatments for this debilitating condition. **Importance:** This is arguably the most significant unmet need in COVID-19 treatment. Millions worldwide suffer from long COVID, severely impacting their quality of life and healthcare systems. Unlocking effective treatments for PASC would dramatically improve the lives of a substantial population.

2. Developing Broad-Spectrum Antivirals with High Efficacy and Accessibility. Definition: Research into novel antiviral drugs that target common viral processes, effective against current and future SARS-CoV-2 variants, and potentially other coronaviruses. Prioritizing oral formulations, low cost, and ease of distribution. **Importance:** While current antivirals like Paxlovid are effective, there's a need for more widely accessible, even more potent, and resistance-proof options to treat acute infection, especially in early stages and for vulnerable populations. This also prepares for future pandemics.

3. Optimizing Treatment Strategies for Critically Ill Patients and Preventing Progression to Severe Disease. Definition: Continued research on immunomodulatory therapies (e.g., corticosteroids, IL-6 inhibitors), anticoagulants, and other interventions to manage severe COVID-19, reduce mortality, and prevent the need for intensive care and mechanical ventilation. This includes identifying optimal timing and patient subgroups for these treatments. **Importance:** While treatments for severe disease have improved, reducing mortality and preventing progression to critical illness remains vital, particularly for high-risk individuals.

4. Identifying Biomarkers for Disease Severity, Prognosis, and Treatment Response. Definition: Discovery and validation of biological markers (e.g., in blood, urine, or imaging) that can accurately predict who will develop severe disease, who is at risk for long COVID, and who will respond best to specific treatments. **Importance:** Biomarkers would enable personalized medicine, allowing clinicians to intervene early with targeted therapies, avoiding unnecessary treatments for those unlikely to benefit, and guiding resource allocation.

5. Understanding the Immunopathology of SARS-CoV-2 Infection and Host Response. Definition: Deep dives into how SARS-CoV-2 interacts with the human immune system, the mechanisms of immune evasion, the causes of dysregulated immune responses (e.g., cytokine storm), and how these contribute to disease severity and long-term complications. **Importance:** A thorough understanding of the immune response is fundamental to developing effective therapeutics, vaccines, and strategies to prevent pathology.

6. Developing Targeted Therapies for Specific Organ Damage (Lungs, Heart, Brain, Kidneys). Definition: Research focused on treatments that specifically address the pathology in organs commonly affected by severe COVID-19, such as lung fibrosis, myocarditis, neurological damage, and kidney injury. **Importance:** Beyond general antiviral or anti-inflammatory approaches, organ-specific therapies are needed to mitigate long-term damage and improve recovery.

7. Addressing Health Disparities in COVID-19 Treatment and Outcomes. Definition: Research to understand and address the socioeconomic, racial, ethnic, geographic, and other factors that lead to unequal access to diagnosis and treatment, and disproportionate disease burden and mortality in certain populations.

Importance: Ensuring equitable access to effective treatments and better outcomes for all populations is a critical ethical and public health imperative.

8. Understanding Viral Evolution and Developing Variant-Proof Therapies. Definition: Ongoing surveillance of SARS-CoV-2 mutations, research into how these mutations impact transmissibility, virulence, and susceptibility to existing treatments, and the development of therapies (e.g., broadly neutralizing antibodies, pan-coronavirus antivirals) that remain effective against emerging variants. **Importance:** SARS-CoV-2 continues to evolve, necessitating vigilance and treatments that are robust against future variants.

9. Developing Rapid, Accurate, and Accessible Diagnostic Technologies for Early Treatment. Definition: Research into new point-of-care tests that can quickly and accurately diagnose COVID-19 and identify individuals at high risk of severe disease, enabling timely administration of early treatments. **Importance:** Early diagnosis is key to maximizing the effectiveness of antiviral therapies and allowing prompt initiation of care.

10. Improving Rehabilitation and Recovery Programs for Post-COVID Patients. Definition: Research into effective rehabilitation strategies (physical, occupational, cognitive, psychological) for individuals recovering from severe COVID-19 and those with long COVID, aiming to improve functional recovery and quality of life. **Importance:** Comprehensive rehabilitation is essential for restoring function and well-being after a serious illness or with chronic symptoms.

11. Investigating Repurposed Drugs and Combinatorial Therapies. Definition: Systematic evaluation of existing drugs for their potential efficacy against SARS-CoV-2 or its associated pathology, and research into optimal combinations of different therapeutic agents to achieve synergistic effects and overcome resistance. **Importance:** Repurposing drugs can accelerate the availability of new treatments, and combination therapies often provide broader efficacy and mitigate resistance.

12. Understanding the Role of the Microbiome in COVID-19 Severity and Recovery. Definition: Research into how the composition of the gut and respiratory microbiomes influences susceptibility to infection, disease severity, and recovery, and exploring potential microbiome-based therapeutic interventions. **Importance:** The microbiome is an emerging area with potential to reveal new therapeutic targets or strategies.

13. Developing Advanced Delivery Methods for Therapeutics (e.g., Inhaled Therapies). Definition: Research into innovative ways to deliver drugs directly to the site of infection (e.g., lungs via inhalation), potentially increasing efficacy and reducing systemic side effects. **Importance:** Targeted delivery could significantly improve the therapeutic index of many treatments.

14. Addressing Mental Health Impacts of COVID-19 and its Treatment. Definition: Research into the prevalence, mechanisms, and effective interventions for mental health conditions (e.g., anxiety, depression, PTSD) in both acute and long COVID patients, and healthcare workers. **Importance:** The pandemic has had profound mental health consequences, and addressing these is crucial for holistic recovery.

15. Understanding Pediatric COVID-19 and PASC. Definition: Specific research focused on the unique aspects of SARS-CoV-2 infection, acute disease, and PASC in children and adolescents, including multisystem inflammatory syndrome in children (MIS-C). **Importance:** Children represent a distinct population with different disease presentations and long-term sequelae that require dedicated study.

16. Nutritional and Metabolic Support in COVID-19. Definition: Research into optimal nutritional strategies and metabolic interventions to support immune function, reduce inflammation, and aid recovery in patients with acute or long COVID. **Importance:** Nutrition plays a vital role in patient resilience and recovery from illness.

17. Refining Ventilation and Respiratory Support Strategies. Definition: Ongoing research into optimal mechanical ventilation settings, non-invasive respiratory support, and adjunctive therapies to improve outcomes for patients with acute respiratory distress syndrome (ARDS) due to COVID-19. **Importance:** While foundational, continuous improvement in critical care support remains important for the sickest patients.

18. Understanding Co-infections and Their Impact on COVID-19 Severity and Treatment. Definition: Research into the prevalence and impact of bacterial, fungal, and other viral co-infections in COVID-19 patients, and optimal diagnostic and treatment strategies for these co-infections. **Importance:** Co-infections can complicate COVID-19, leading to worse outcomes, and require specific management.

19. Enhancing Telemedicine and Remote Monitoring for COVID-19 Patients. Definition: Research into the effectiveness and optimal implementation of remote patient monitoring technologies and telemedicine platforms for managing COVID-19 patients (both acute and long COVID) at home, reducing healthcare burden and improving access to care. **Importance:** Remote care can improve patient access, reduce hospitalizations, and optimize resource utilization.

20. Developing Robust Clinical Trial Infrastructure for Rapid Pandemic Response. Definition: Research and development of adaptive clinical trial designs, international collaboration mechanisms, and streamlined regulatory processes to enable rapid and efficient testing of new therapeutics during future pandemics. **Importance:** Learning from the COVID-19 pandemic to build more agile research capabilities is crucial for future health crises.

Perplexity 1-20 are as follows:

1. Developing Effective Treatments for Long COVID. Advancing pharmacological and non-pharmacological therapies for Long COVID, understanding its pathophysiology, and identifying reliable biomarkers for diagnosis and management.

2. Accelerating Antiviral and Immunomodulatory Drug Development. Discovering and testing new antivirals, immunomodulators, and combination therapies to treat acute and severe COVID-19, including emerging variants.

3. Vaccine Innovation and Optimization. Improving vaccine efficacy, durability, and delivery, including development of next-generation vaccines and strategies for vulnerable populations.

4. Understanding and Managing COVID-19 Variants. Investigating the evolution, transmission, and clinical impact of SARS-CoV-2 variants to inform treatment and prevention strategies.

5. Early Detection and Rapid Diagnostics. Developing rapid, accurate, and accessible diagnostic tools for early identification and monitoring of infection and disease progression.

6. Addressing Drug Resistance and Treatment Failure. Monitoring and preventing resistance to antivirals and immunotherapies, and designing strategies to overcome treatment failure.

7. Optimizing Clinical Management Across Populations. Tailoring treatment protocols for diverse groups, including children, elderly, pregnant individuals, and those with comorbidities.

8. Integrating COVID-19 Care with Other Health Services. Researching models for integrating COVID-19 treatment with other essential health services, especially in resource-limited settings.

9. Preventing and Treating Severe and Critical Illness. Improving interventions for severe respiratory failure, multi-organ dysfunction, and complications such as thrombosis.

10. Health Systems Strengthening and Preparedness. Enhancing healthcare capacity, resource allocation, and preparedness for current and future waves or pandemics.

11. Reducing Health Inequities in Treatment Access. Investigating and addressing disparities in access to COVID-19 treatments and outcomes across socioeconomic, racial, and geographic groups.

12. Infection Prevention and Control in Healthcare Settings. Developing and evaluating strategies to protect healthcare workers and prevent nosocomial transmission.

13. Understanding Long-Term Outcomes and Survivorship. Studying the long-term physical, psychological, and social impacts of COVID-19 and its treatments.

14. Enhancing Community Engagement and Public Trust. Researching effective communication, community engagement, and strategies to combat misinformation and improve adherence to treatment and prevention.

15. Improving Mental Health Support for Patients and Providers. Addressing the psychological effects of COVID-19 and its treatment on patients, families, and healthcare workers.

16. Pediatric COVID-19 Treatment and Prevention. Advancing research into optimal therapies and prevention strategies for children and adolescents.

17. Optimizing Treatment for High-Risk and Immunocompromised Patients. Developing tailored approaches for those with underlying health conditions or compromised immunity.

18. Integration of Digital Health and AI in Treatment Delivery. Leveraging digital tools, artificial intelligence, and telemedicine to improve diagnosis, monitoring, and personalized care.

19. Socioeconomic and Policy Research. Studying the socioeconomic determinants of treatment access and outcomes, and evaluating the impact of public health policies.

20. Preparing for Future Pandemics. Building on COVID-19 research to strengthen global surveillance, rapid response, and R&D infrastructure for future emerging infectious diseases.

A4: HIV

ChatGPT 1-20 are as follows:

1. HIV Cure Research (Sterilizing and Functional Cure). Goal: Eliminate the virus from the body (sterilizing) or achieve drug-free control (functional). **Why it matters**: Lifelong therapy is burdensome and costly; a cure is transformative. **Example**: Latency-reversing agents (LRAs), CRISPR-based editing, stem cell transplant studies.

2. HIV Reservoir Characterization and Eradication. Goal: Understand and eliminate latent HIV reservoirs (e.g., in CD4+ T cells). **Why it matters**: These hidden viral stores reignite infection if treatment stops. **Example**: Single-cell studies of reservoir cells, tissue mapping of viral persistence.

3. Long-Acting Antiretroviral Therapy (LA-ART) Optimization. Goal: Develop and improve extended-duration HIV drugs (monthly to yearly). **Why it matters**: Reduces pill burden, stigma, and adherence challenges. **Example**: Injectable cabotegravir/rilpivirine; nanocarriers; implants.

4. Development of Broadly Neutralizing Antibodies (bNAbs). Goal: Harness antibodies that neutralize diverse HIV strains for therapy or prevention. **Why it matters**: Can delay or replace ART and may assist in cure strategies. **Example**: VRC01-class antibodies in passive immunization trials.

5. Immune-Based Therapies and Immune Modulation. Goal: Strengthen immune control over HIV, especially post-ART. **Why it matters**: Immune exhaustion contributes to poor control and complications. **Example**: Therapeutic vaccines, checkpoint inhibitors (PD-1/CTLA-4), TLR agonists.

6. Improved Strategies for Early HIV Detection and Treatment. Goal: Diagnose and initiate treatment during acute/early infection. **Why it matters**: Early ART reduces reservoir size and immune damage. **Example**: Point-of-care rapid tests, HIV RNA screening in high-risk settings.

7. Integration of HIV and STI Care. Goal: Co-manage HIV with other sexually transmitted infections. **Why it matters**: STIs increase HIV transmission risk and treatment complexity. **Example**: Multiplex diagnostic platforms, integrated clinics, partner services.

8. Comorbidity Management in People with HIV (PWH). Goal: Address non-HIV diseases (e.g., cardiovascular, liver, kidney, neurocognitive). **Why it matters**: PWH face high burden of age-related and ART-related complications. **Example**: Statins for inflammation, cognitive rehabilitation, cancer screening.

9. Personalized and Precision HIV Therapy. Goal: Tailor ART based on host and viral genetics, pharmacogenomics, and resistance. **Why it matters**: Maximizes efficacy, reduces side effects, and avoids resistance. **Example**: HLA testing, resistance genotyping, therapeutic drug monitoring.

10. Resistance Prevention and Management. Goal: Understand and prevent antiretroviral resistance. **Why it matters**: Resistance leads to treatment failure and transmission of drug-resistant HIV. **Example**: Surveillance of resistant strains, AI-based regimen selection tools.

11. HIV in Key Populations. Goal: Improve care for MSM, transgender people, sex workers, PWID, incarcerated populations. **Why it matters**: These groups face disproportionate HIV burden and stigma. **Example**: Culturally tailored outreach, decentralized PrEP/ART models.

12. Pediatric and Adolescent HIV Care. Goal: Develop safe, effective, age-appropriate formulations and care models. **Why it matters**: Pediatric treatment lags behind adults; adolescents have adherence issues. **Example**: Dispersible tablets, long-acting injectables, youth-centered clinics.

13. Integration of Mental Health and Substance Use Services. Goal: Address depression, trauma, and addiction in HIV care. **Why it matters**: Mental health and substance use are major barriers to adherence and engagement. **Example**: On-site counseling, MAT programs, trauma-informed care.

14. HIV Cure-Related Biomarker Discovery. Goal: Identify biomarkers that predict reservoir size, immune control, or cure potential. **Why it matters**: Needed for tracking progress in cure trials and personalizing strategies. **Example**: Cell-associated HIV RNA/DNA, T-cell activation markers.

15. Vaccine-Based Therapeutic Approaches. Goal: Use vaccines to enhance immune control in infected individuals. **Why it matters**: Could replace or complement ART in maintaining viral suppression. **Example**: DNA/mRNA vaccines targeting conserved HIV epitopes.

16. Optimization of PrEP to Prevent Superinfection in Treated Individuals. Goal: Prevent reinfection with different HIV strains, especially drug-resistant ones. **Why it matters**: Superinfection complicates treatment and increases public health risk. **Example**: Dual protection strategies, risk counseling.

17. Novel Drug Delivery Systems. Goal: Improve adherence and access via innovative delivery platforms. **Why it matters**: Better delivery improves efficacy and accessibility. **Example**: Microneedle patches, oral rings, AI-powered pill adherence tools.

18. Addressing HIV-Associated Inflammation and Aging. Goal: Mitigate persistent immune activation and its effects on aging. **Why it matters**: Even with ART, chronic inflammation accelerates aging and organ damage. **Example**: Anti-inflammatory drugs, senolytics, gut microbiome therapies.

19. Global Health Equity and Implementation Science. Goal: Translate research into scalable, equitable care in low-resource settings. **Why it matters**: Disparities in access to treatment drive global mortality. **Example**: Differentiated care models, community-based ART delivery.

20. Digital Health and mHealth Integration. Goal: Use technology to support adherence, monitoring, and engagement. **Why it matters**: Can boost outcomes in hard-to-reach or disengaged populations. **Example**: SMS adherence reminders, telemedicine, AI chatbots.

Claude 1-20 are as follows:

1. HIV Cure Research Developing strategies to achieve a sterilizing cure or functional cure, including "shock and kill" approaches, gene editing technologies like CRISPR, and immune-based interventions. This represents the ultimate goal of HIV research.

2. Long-Acting Antiretroviral Therapies Creating injectable and implantable formulations that provide months of protection, improving adherence and quality of life. This includes both treatment and prevention applications.

3. Broadly Neutralizing Antibody Development Advancing bnAbs for both treatment and prevention, including passive immunization strategies and methods to induce bnAb production through vaccination or gene therapy.

4. HIV Vaccine Development Pursuing both prophylactic and therapeutic vaccines using novel approaches including mosaic vaccines, germline targeting, and structure-based design to overcome HIV's genetic diversity.

5. Latent Reservoir Characterization Understanding HIV persistence mechanisms, reservoir location and dynamics, and developing methods to measure and target latent HIV in different anatomical compartments.

6. Pre-Exposure Prophylaxis (PrEP) Innovation Developing new PrEP modalities including long-acting injectables, vaginal rings, microbicides, and combination prevention strategies to expand access and effectiveness.

7. Drug Resistance Prevention and Management Researching resistance mechanisms, developing resistance-barrier drugs, and creating personalized treatment strategies based on resistance testing and viral genomics.

8. Pediatric HIV Research Developing age-appropriate formulations, optimal treatment regimens for children, and strategies to prevent mother-to-child transmission in resource-limited settings.

9. HIV and Aging Research Investigating age-related comorbidities, drug interactions, and optimal care strategies for the growing population of older adults with HIV.

10. Immune Reconstitution and Inflammation Understanding persistent immune dysfunction despite viral suppression, chronic inflammation, and developing interventions to restore immune function and reduce inflammation.

11. Central Nervous System HIV Researching HIV-associated neurocognitive disorders, developing drugs that penetrate the blood-brain barrier, and understanding brain reservoir dynamics.

12. Co-infection Management Optimizing treatment for HIV co-infections including tuberculosis, hepatitis B/C, and other opportunistic infections, particularly in resource-limited settings.

13. HIV Prevention in Key Populations Developing targeted interventions for high-risk groups including men who have sex with men, sex workers, people who inject drugs, and transgender individuals.

14. Tissue Reservoir Research Investigating HIV persistence in sanctuary sites including the gut, genital tract, and lymphoid tissues, and developing strategies to target these reservoirs.

15. Novel Therapeutic Targets Exploring new drug targets beyond traditional reverse transcriptase, protease, and integrase inhibitors, including host factors and novel viral targets.

16. Pharmacokinetics and Drug Optimization Improving drug penetration into tissues, reducing drug interactions, and optimizing dosing strategies for different populations and co-medications.

17. HIV Transmission Research Understanding transmission dynamics, developing better models to predict transmission risk, and creating more effective prevention strategies.

18. Global Health Implementation Researching optimal strategies for HIV care delivery in resource-limited settings, including task-shifting, point-of-care testing, and community-based interventions.

19. Mental Health and Substance Use Addressing the high prevalence of mental health disorders and substance use among people with HIV, and their impact on treatment adherence and outcomes.

20. HIV Stigma and Discrimination Research Investigating the impact of stigma on health outcomes and developing interventions to reduce stigma and discrimination in healthcare settings and communities.

DeepSeek 1-20 are as follows:

1. HIV Cure Strategies. **Eradication:** Eliminate latent viral reservoirs (e.g., "shock and kill," gene editing). **Functional Cure:** Achieve long-term remission without ART (e.g., CCR5Δ32 modifications).

2. Long-Acting Antiretrovirals (ARVs). Develop ultra-long-acting injectables/implants (e.g., yearly formulations). Improve adherence-free prevention/treatment (e.g., cabotegravir + rilpivirine).

3. Broadly Neutralizing Antibodies (bNAbs). Enhance potency and durability of bNAbs for treatment/prevention. Overcome viral escape mutations via engineered multi-specific antibodies.

4. Preventive Vaccines. Develop an effective prophylactic vaccine (e.g., mRNA-based approaches). Target conserved epitopes to combat HIV diversity.

5. Reservoir Detection & Measurement. Improve assays to quantify latent HIV (e.g., DNA/RNA sequencing, PET imaging). Identify biomarkers predicting rebound after ART interruption.

6. Immune-Based Therapies. Restore CD4+ T-cell function (e.g., IL-2, PD-1 blockade). Enhance innate immunity (e.g., NK cell therapies).

7. Pediatric HIV Cure & Treatment. Optimize ART for neonates to prevent reservoir establishment. Develop child-friendly long-acting formulations.

8. Targeting Viral Persistence Mechanisms. Block clonal expansion of infected cells. Disrupt HIV integration sites (e.g., CRISPR/dCas9).

9. Comorbidities & Aging with HIV. Address accelerated aging, neurocognitive decline, and cardiovascular disease. Reduce chronic inflammation despite viral suppression.

10. Drug Resistance Solutions. Develop ARVs with higher genetic barriers to resistance. Create salvage regimens for multi-drug-resistant HIV.

11. Mucosal & Tissue-Specific Therapies. Target gut-associated lymphoid tissue (GALT) reservoirs. Develop topical PrEP/PEP agents (e.g., vaginal rings).

12. Gene & Cell Therapy. CCR5 gene editing (e.g., CRISPR, zinc finger nucleases). CAR-T cells engineered to target HIV-infected cells.

13. Therapeutic Vaccines. Boost immune control post-ART (e.g., dendritic cell vaccines). Combine vaccines with latency-reversing agents.

14. Simplifying Treatment Regimens. Develop once-weekly oral pills or single-drug therapies. Reduce pill burden for adherence-challenged populations.

15. Neuro-HIV & CNS Penetration. Improve ART penetration into the brain to combat HIV-associated neurocognitive disorders (HAND). Target viral reservoirs in the CNS.

16. Pre-Exposure Prophylaxis (PrEP) Innovations. Expand options beyond oral Truvada/Descovy (e.g., on-demand PrEP). Develop non-antiretroviral PrEP (e.g., bNAbs, microbicides).

17. Post-Treatment Control. Identify "elite controllers" mechanisms for drug-free remission. Test immunotherapy combinations to sustain viral suppression.

18. Global Access & Equity. Reduce cost of advanced therapies (e.g., gene editing, bNAbs). Improve diagnostics/treatment in low-resource settings.

19. Co-Infections (TB, Hepatitis, etc.). Optimize HIV/TB co-treatment to reduce drug interactions. Cure HBV/HCV in HIV-coinfected patients.

20. Behavioral & Structural Interventions. Integrate PrEP/ART with harm reduction programs. Combat stigma to improve testing/treatment adherence.

Gemini 1-20 are as follows:

1. Developing a Safe, Effective, and Scalable HIV Cure (Eradication or Functional Cure). Definition: This encompasses research into strategies to permanently eliminate the HIV virus from the body (sterilizing cure) or to achieve long-term remission without the need for antiretroviral therapy (ART) (functional cure). Key areas include gene editing, "shock and kill" (reactivating latent virus and then clearing infected cells), "block and lock" (permanently silencing the virus), and therapeutic vaccines. **Importance:** This is the ultimate goal. Lifelong ART, while effective, carries a significant burden, including daily medication, potential side effects, and the psychological impact of living with HIV. A cure would eliminate this burden and the need for continuous medical care, transforming the lives of millions.

2. Eradicating or Controlling the HIV Latent Reservoir. Definition: The primary barrier to an HIV cure is the latent reservoir – a small population of infected cells where the virus lies dormant, undetectable by current treatments, and ready to reactivate if ART is stopped. Research focuses on precisely locating, characterizing, and targeting these cells. **Importance:** This is inextricably linked to the cure. Without addressing the latent reservoir, sustained ART-free remission or eradication is impossible. Understanding its formation, persistence, and reactivation is fundamental.

3. Developing Long-Acting Antiretroviral Therapies (LA-ART). Definition: Research into new formulations of ART that require less frequent dosing (e.g., injections every few months, implants, patches). This also includes optimizing the pharmacokinetics and safety of these long-acting agents. **Importance:** Daily oral pills, even with excellent efficacy, can be a major barrier to adherence for many, due to "pill fatigue," stigma, or logistical challenges. LA-ART offers a revolutionary approach to improve adherence, convenience, privacy, and ultimately, treatment outcomes, particularly in resource-limited settings.

4. Developing a Safe and Effective HIV Vaccine. Definition: Research and development of both prophylactic (preventative) vaccines to stop new infections and therapeutic vaccines to help control the virus in people already living with HIV. This involves understanding the immune responses needed for protection and overcoming HIV's remarkable ability to mutate and evade the immune system. **Importance:** A vaccine would be a game-changer for prevention, particularly in high-incidence populations, and could potentially contribute to functional cures by boosting immune control in treated individuals.

5. Understanding and Mitigating HIV-Associated Non-AIDS (NADS) Conditions. Definition: Even with effective ART, people living with HIV are at increased risk of age-related comorbidities like cardiovascular disease, kidney disease, bone disease, neurocognitive disorders, and certain cancers (non-AIDS defining cancers). Research aims to understand the mechanisms behind these conditions (e.g., chronic inflammation, ART toxicity, co-infections) and develop strategies to prevent or manage them. **Importance:** As people with HIV live longer, improving their overall health and quality of life is paramount. NADs significantly impact morbidity and mortality in the ART era.

6. Addressing and Overcoming Antiretroviral Drug Resistance. Definition: Research into the genetic mutations that allow HIV to become resistant to existing ART drugs, developing new drug classes with novel mechanisms of action, and designing treatment strategies (e.g., combination therapies) that minimize the development of resistance. **Importance:** Drug resistance can lead to treatment failure, requiring complex and often more toxic rescue regimens. Maintaining effective drug options is crucial for long-term viral suppression.

7. Early Detection and Rapid Initiation of Treatment (Test-and-Treat Strategies). Definition: Research into developing more accessible, affordable, and rapid diagnostic tests, particularly for point-of-care settings,

and implementing strategies to link newly diagnosed individuals to ART as quickly as possible. **Importance:** Early diagnosis and immediate treatment improve individual health outcomes, prevent disease progression, and significantly reduce HIV transmission.

8. Personalized Treatment Approaches and Biomarker Development. Definition: Utilizing individual patient data (genetics, viral characteristics, immune status, comorbidities) to tailor ART regimens for optimal efficacy and minimal side effects. This includes identifying biomarkers that predict treatment response, risk of side effects, or progression of NADs. **Importance:** "One-size-fits-all" approaches may not be optimal for everyone. Personalizing treatment can maximize benefits and reduce the burden on patients.

9. Understanding the Role of Inflammation and Immune Dysfunction. Definition: Investigating the causes and consequences of chronic inflammation and immune activation that persist in people with HIV despite viral suppression, and how these contribute to NADs and HIV persistence. Developing anti-inflammatory or immune-modulating therapies. **Importance:** Chronic inflammation is a key driver of many HIV-associated comorbidities.

10. Improving Prevention Technologies and Their Uptake. Definition: While not directly *treatment*, advancements in prevention (e.g., next-generation PrEP, microbicides, multipurpose prevention technologies) directly reduce the number of people who need treatment. Research on implementation science is also critical to ensure these tools reach those who need them most. **Importance:** Preventing new infections lessens the overall burden of disease and the demand on treatment systems.

11. Addressing Stigma and Discrimination and Their Impact on Treatment Adherence and Outcomes. Definition: Research into the social and behavioral factors that contribute to HIV-related stigma, its impact on testing, linkage to care, adherence, and quality of life, and developing effective interventions to reduce stigma at individual, community, and systemic levels. **Importance:** Stigma is a major non-biological barrier to effective treatment and prevention globally.

12. Optimizing Treatment for HIV-Infected Children and Adolescents. Definition: Developing age-appropriate ART formulations, understanding the long-term effects of HIV and ART on growing bodies and developing brains, and addressing the unique adherence challenges faced by this population. **Importance:** Children and adolescents living with HIV have specific needs that require tailored research.

13. Improving HIV Care in Resource-Limited Settings. Definition: Research into simplified treatment regimens, cost-effective diagnostics, decentralized care models, task-shifting, and supply chain management to ensure equitable access to high-quality HIV care in low- and middle-income countries. **Importance:** The majority of people living with HIV reside in these settings, and sustainable, accessible care is critical.

14. Understanding and Treating Neurocognitive Disorders. Definition: Investigating the mechanisms by which HIV affects the central nervous system, leading to cognitive impairment (HAND - HIV-Associated Neurocognitive Disorder), and developing effective treatments and interventions to preserve brain health. **Importance:** HAND significantly impacts quality of life and functional independence.

15. Addressing Mental Health Co-morbidities. Definition: Research into the prevalence, mechanisms, and effective interventions for mental health conditions (e.g., depression, anxiety, substance use disorders) that are highly prevalent among people living with HIV. **Importance:** Mental health is integral to overall well-being and impacts ART adherence.

16. Nutritional Interventions and Management of Wasting Syndrome. Definition: Research into effective nutritional support, dietary interventions, and pharmacological approaches to prevent and treat HIV-associated wasting and malnutrition. **Importance:** Malnutrition impacts immune function, treatment tolerance, and overall health.

17. Improving Monitoring and Diagnostics for Treatment Effectiveness. Definition: Developing simpler, cheaper, and more accessible viral load tests, resistance testing, and other biomarkers to monitor treatment response and identify treatment failure earlier, particularly in settings with limited laboratory infrastructure. **Importance:** Effective monitoring is essential for guiding treatment decisions and optimizing outcomes.

18. Research on Aging with HIV. Definition: As people with HIV live longer, understanding the unique aspects of aging with chronic HIV infection, including accelerated aging processes, polypharmacy, and the accumulation of comorbidities, and developing age-specific care guidelines. **Importance:** This is a growing population with distinct clinical needs.

19. Exploring Gene Therapy and Cell-Based Therapies Beyond Stem Cell Transplants. Definition: Investigating approaches to modify a person's own cells (e.g., T-cells) to make them resistant to HIV infection or to enhance their ability to clear infected cells, moving beyond the complex and risky stem cell transplantation

approach. **Importance:** Offers potential for highly targeted and durable interventions, especially in cure research.

20. Developing Novel Animal Models for HIV Research. Definition: Creating and refining animal models (e.g., humanized mice, non-human primates) that more accurately mimic human HIV infection, disease progression, and response to treatment, enabling more effective preclinical testing of new therapies and cure strategies. **Importance:** Robust animal models are crucial for advancing basic science and translating findings into clinical trials.

Perplexity 1-20 are as follows:

1. Achieving a Functional or Sterilizing Cure. Research aimed at sustained viral remission or complete eradication of HIV from the body, including strategies to target latent reservoirs and innovative gene therapies.

2. Developing Next-Generation Antiretroviral Therapies (ART). Creating safer, more effective, and easier-to-use ART regimens, including long-acting formulations and therapies that reduce side effects and pill burden.

3. Understanding and Targeting HIV Reservoirs. Defining, measuring, and eliminating the sources of persistent HIV in the body, including improving assays and imaging to track reservoir size and activity.

4. Optimizing ART for All Populations. Ensuring ART safety and efficacy for diverse groups, including children, adolescents, pregnant and lactating women, and people with comorbidities.

5. Addressing HIV-Associated Comorbidities and Coinfections. Research to prevent, diagnose, and manage conditions such as tuberculosis, hepatitis, and non-communicable diseases in people living with HIV.

6. Enhancing Early Detection and Diagnosis. Developing and deploying sensitive, specific, and accessible diagnostic tools, including point-of-care and at-home viral load monitoring.

7. Improving Long-Term Adherence and Retention in Care. Innovations to support lifelong adherence to therapy, including behavioral interventions, digital health tools, and long-acting injectable ART.

8. Preventing and Managing Drug Resistance. Monitoring, understanding, and developing strategies to prevent or overcome resistance to ART and other HIV treatments.

9. Pediatric HIV Treatment and Prevention. Advancing research into optimal drug formulations, dosing, and strategies for infants, children, and adolescents, including mother-to-child transmission prevention.

10. Integration of HIV and Other Health Services. Research on models to deliver integrated care for HIV alongside other infectious and non-infectious diseases, mental health, and reproductive health.

11. Social, Behavioral, and Ethical Research. Understanding and addressing stigma, discrimination, and barriers to care, and ensuring ethical conduct in HIV research and treatment delivery.

12. Advancing HIV Vaccine and Immunotherapy Research. Supporting the development of preventive and therapeutic vaccines, as well as immune-based therapies to control or eradicate HIV.

13. Implementation Science and Health Systems Strengthening. Studying how to best deliver proven interventions at scale, especially in low- and middle-income countries, and closing gaps in the care cascade.

14. Personalized and Precision Medicine Approaches. Tailoring HIV treatment based on genetic, immunological, and virological profiles to improve outcomes and minimize side effects.

15. Reducing HIV-Related Stigma and Discrimination. Researching interventions to reduce stigma and its impact on testing, treatment uptake, and quality of life for people living with HIV.

16. Addressing Gender and Key Population Disparities. Developing targeted strategies for women, young people, LGBTQ+ communities, sex workers, people who inject drugs, and other vulnerable groups.

17. Optimizing Treatment for HIV/TB Coinfection. Improving diagnostics, treatment regimens, and care integration for people with both HIV and tuberculosis, especially in high-burden settings.

18. Enhancing Community Engagement and Participation. Involving people living with HIV and affected communities in research design, implementation, and dissemination.

19. Advancing Cell and Gene Therapies. Exploring gene editing, stem cell, and other advanced therapies as potential curative or remission-inducing interventions.

20. Monitoring and Improving Long-Term Outcomes and Survivorship. Research into the long-term effects of HIV and its treatment, including aging, quality of life, and chronic disease management.

Part B: Texts for over time analysis

B1: Alzheimer’s disease

DeepSeek

1. Early Detection & Biomarkers. Develop blood tests (e.g., p-tau217, GFAP) and retinal/cognitive digital biomarkers to identify AD 5–10 years before symptoms.

2. Disease-Modifying Therapies (DMTs) Beyond Amyloid. Target tau pathology, neuroinflammation, and synaptic dysfunction (e.g., tau vaccines, TREM2 agonists).

3. Precision Medicine Approaches.Stratify patients by genetic (APOE4, TRE M2), proteomic, and metabolic subtypes for tailored therapies.

4. Amyloid Clearance Optimization. Improve anti-amyloid immunotherapies (e.g., lecanemab, donanemab) to maximize efficacy while minimizing ARIA (brain swelling/bleeding).

5. Tau-Targeted Therapies. Advance tau aggregation inhibitors, anti-tau antibodies, and microtubule stabilizers to halt neurodegeneration.

6. Neuroinflammation Modulation. Target microglial dysfunction (e.g., TREM2, NLRP3 inflammasome) to reduce neuronal damage.

7. APOE4 Therapeutics. Develop APOE4-to-APOE2/3 converters, gene therapy, or small molecules to mitigate APOE4’s toxic effects.

8. Synaptic Repair & Neuroprotection. Boost BDNF, NMDA receptor modulators, and mitochondrial function to preserve cognition.

9. Combination Therapies. Test amyloid + tau + anti-inflammatory drug combos to address multiple pathways simultaneously.

10. Vascular & Metabolic Interventions. Optimize blood-brain barrier repair, insulin sensitizers, and hypertension control to reduce AD risk.

11. Gut-Brain Axis & Microbiome. Explore probiotics, dietary interventions, and short-chain fatty acids to modulate neuroinflammation.

12. Non-Invasive Brain Stimulation. Refine transcranial magnetic stimulation (TMS) and focused ultrasound to enhance cognitive function.

13. Cognitive Resilience Mechanisms. Study super-agers and cognitive reserve to identify protective factors (e.g., exercise, education).

14. Epigenetic Therapies. Investigate HDAC inhibitors, DNA methylation modulators to reverse age-related gene dysregulation.

15. Exosome-Based Drug Delivery. Engineer nanoparticles/exosomes to cross the BBB and deliver therapeutics (e.g., siRNA, growth factors).

16. Senolytics & Aging Biology. Test senolytic drugs (e.g., dasatinib + quercetin) to clear senescent glial cells driving inflammation.

17. Alternative Splicing & RNA Therapies. Correct splicing errors (e.g., in APP, tau) using antisense oligonucleotides (ASOs).

18. Circadian Rhythm Restoration. Improve sleep therapies (e.g., orexin antagonists) to reduce amyloid/tau accumulation.

19. Psychedelics & Neuroplasticity. Explore low-dose psilocybin, LSD, or 5-HT2A agonists to enhance synaptic connectivity.

20. Real-World Data & Digital Monitoring. Leverage wearables, voice AI, and eye-tracking for early decline detection and trial enrichment.

Perplexity

1. Development of Disease-Modifying Therapies and a Cure. Discover and test interventions that halt, slow, or reverse the underlying pathology of Alzheimer’s disease, such as targeting amyloid, tau, neuroinflammation, and synaptic loss.
2. Early Detection and Diagnosis. Improve and expand sensitive, accessible biomarkers and digital tools for preclinical and early-stage diagnosis to enable timely intervention.
3. Understanding and Addressing Disease Heterogeneity. Study molecular subtypes, mixed dementias, and individual variability to guide precision medicine approaches.
4. Prevention and Risk Reduction Strategies. Identify and validate interventions (pharmacologic and non-pharmacologic) that reduce incidence based on modifiable risk factors at the population and individual level.
5. Elucidating Mechanisms of Disease Pathogenesis. Deepen understanding of the cellular, genetic, and environmental mechanisms driving Alzheimer’s, including the exposome and gene-environment interactions.
6. Development and Validation of Biomarkers. Create robust, scalable biomarkers for diagnosis, prognosis, and monitoring therapeutic response, including imaging and fluid-based markers.
7. Understanding and Managing Comorbidities. Investigate interactions with vascular disease, diabetes, depression, and other comorbidities to optimize care and outcomes.
8. Combination Therapy Approaches. Develop and test multi-targeted interventions to address the multifactorial nature of Alzheimer’s disease.
9. Advancing Precision Medicine. Identify participant subgroups likely to benefit from specific interventions (genetic, molecular, lifestyle) and apply individualized care strategies.

10. Clinical Trials Innovation. Use real-world data, adaptive trial designs, and decentralized research platforms to accelerate evaluation of new interventions, including those repurposed from other conditions.

11. Health Disparity and Diverse Population Research. Address gaps in research and care for underserved and disproportionately affected communities, focusing on health equity and social determinants.

12. Improving Care Models and Patient Quality of Life. Develop and test care interventions, systems, and technologies to maintain function, independence, and well-being for patients and caregivers.

13. Non-Pharmacological Therapies. Rigorously evaluate lifestyle, cognitive, physical, and psychosocial therapies for efficacy across disease stages.

14. Real-World Evidence and Implementation Science. Integrate findings from clinical settings to refine and scale effective interventions nationwide and globally.

15. Resilience, Risk, and Protective Factor Research. Investigate why some individuals remain cognitively healthy despite high-risk profiles or brain pathology, to inform prevention and therapy.

16. Advance Digital Health and Remote Monitoring. Develop and deploy digital tools for symptom tracking, progression monitoring, and adherence support.

17. Supporting and Training the Dementia Care Workforce. Enhance evidence-based training and support for professionals delivering Alzheimer's care.

18. Policy, Economic, and Ethical Research. Study the cost-effectiveness, resource allocation, and ethical considerations of new therapies and care paradigms.

19. Collaboration and Data Sharing. Foster multidisciplinary research, inclusivity, and global data sharing to accelerate progress and avoid duplication.

20. Engagement of People with Lived Experience. Involve patients, families, and caregivers in research prioritization, study design, and implementation to ensure relevance and impact.

B2: Cancer

DeepSeek

1. Early Detection & Liquid Biopsies. Develop highly sensitive, non-invasive tests (e.g., blood-based multi-cancer early detection) to identify tumors at curable stages.

2. Immunotherapy Advancements. Improve checkpoint inhibitors, CAR-T cells, and cancer vaccines to overcome resistance and expand efficacy to "cold" tumors.

3. Precision Oncology & Biomarker Discovery. Expand genomic, proteomic, and metabolomic profiling to match patients with optimal therapies.

4. Overcoming Drug Resistance. Understand and target mechanisms (e.g., tumor heterogeneity, adaptive pathways) that lead to treatment failure.

5. Tumor Microenvironment (TME) Modulation. Develop therapies targeting stroma, immune suppression (e.g., Tregs, MDSCs), and hypoxia to enhance treatment penetration.

6. AI & Computational Oncology. Integrate machine learning for early diagnosis, drug discovery, and personalized treatment optimization.

7. Epigenetic Therapies. Develop drugs targeting DNA methylation, histone modifications, and non-coding RNAs to reverse malignant gene silencing.

8. Metastasis Prevention & Treatment. Uncover mechanisms of dissemination and develop therapies to block metastatic colonization.

9. Cancer Stem Cell Eradication. Target self-renewing, therapy-resistant subpopulations driving recurrence.

10. Microbiome & Cancer Therapy. Harness gut microbiota to improve immunotherapy response and reduce toxicity.

11. Radiotherapy Enhancements. Advance proton therapy, FLASH radiotherapy, and radiosensitizers to spare healthy tissue.

12. Pediatric & Rare Cancer Research. Expand molecular profiling and targeted therapies for underserved populations.

13. Combination Therapy Optimization. Systematically identify synergistic drug-immune-radiation combinations using high-throughput screening.

14. Senescence & Apoptosis Targeting. Develop senolytics and pro-apoptotic drugs to eliminate dormant cancer cells.

15. Cost Reduction & Global Access. Lower costs of biologics (e.g., CAR-T, bispecific antibodies) for low/middle-income countries.

16. Cancer Vaccines (Preventive & Therapeutic). Advance mRNA-based and neoantigen vaccines for high-risk individuals and adjuvant settings.

17. Nanomedicine & Drug Delivery. Improve tumor-targeted delivery (e.g., nanoparticles, antibody-drug conjugates) to reduce off-target effects.

18. PARP Inhibitors & DNA Repair Exploitation. Expand synthetic lethality approaches beyond BRCA-mutant cancers.

19. Real-World Data & Adaptive Trials. Leverage real-world evidence (RWE) and basket trials to accelerate approvals.

20. Survivorship & Late-Effects Management. Develop strategies to mitigate long-term toxicity (e.g., cardiotoxicity, secondary cancers).

Perplexity

1. Early Detection and Diagnosis. Develop more accurate and less invasive methods for early cancer detection, including blood-based and imaging technologies.
2. Precision Oncology and Personalized Treatment. Tailor treatments based on the genetic and molecular characteristics of each patient's cancer, leveraging advances in genomics and precision medicine.
3. Immunotherapy Development. Advance and expand immune-based therapies, such as immune checkpoint inhibitors, CAR-T cells, and cancer vaccines, to more cancer types and stages.
4. Prevention and Risk Reduction. Develop effective strategies to reduce cancer risk through lifestyle, behavioral, and environmental interventions, and expand prevention research for high-risk populations.
5. Understanding and Overcoming Drug Resistance. Study the mechanisms behind resistance to therapies and design strategies or combinations to prevent or overcome resistance.
6. Affordable and Equitable Access to Cancer Therapies. Address disparities in access to care by developing cost-effective treatments and health policies to make advanced therapies available globally, especially in low- and middle-income countries.
7. Cancer Screening Innovation. Improve the sensitivity, specificity, and uptake of cancer screening programs, including for rarer cancers.
8. Minimally Invasive and Targeted Surgery/Radiotherapy. Refine and disseminate image-guided, robot-assisted, and less invasive surgical and radiotherapy techniques to improve outcomes and reduce side effects.
9. Long-term Survivorship and Quality of Life. Find ways to manage long-term physical, mental, and social side effects in cancer survivors, and optimize models of follow-up care.
10. Development of Novel Drug Combinations. Discover and test multi-drug or multi-modal regimens to maximize efficacy and minimize toxicity.
11. Real-World Data and Artificial Intelligence. Harness AI, big data, and machine learning to analyze diagnostics, predict treatment responses, and streamline personalized care pathways.
12. Cancer Microenvironment and Metastasis. Explore how the tumor microenvironment, immune system, and metastatic processes influence tumor progression and therapy response.
13. Research on Cancer in Special Populations. Focus on pediatric cancers, rare cancers, and medically underserved or co-morbid groups to address unique biological and treatment challenges.
14. Improving Outcomes for Advanced and Metastatic Cancer. Develop effective therapies for patients with advanced-stage disease to prolong survival and improve quality of life.
15. Treatment De-escalation and Avoidance of Overtreatment. Identify which patients can benefit from less aggressive therapies to avoid unnecessary harm and improve quality of life.

16. Health Economics and Value-Based Care. Evaluate cost-effectiveness and optimize resource allocation in cancer care, ensuring innovations translate into real-world value.
17. Biomarker Discovery and Validation. Identify markers that predict treatment response, toxicity, relapse, or cure to inform decision-making and clinical trial design.
18. Tumor Heterogeneity and Evolution. Understand intra-tumor genetic diversity and dynamics over time to design adaptive treatment strategies.
19. Translational and Implementation Research. Accelerate the translation of laboratory discoveries into clinical trials and routine practice, optimizing delivery systems for new treatments.
20. Supportive, Palliative, and End-of-Life Care. Advance research on improving symptom management, palliation, and end-of-life decision-making and care models for cancer patients.

B3: COVID-19

DeepSeek

1. Broad-Spectrum Antivirals. Develop pan-coronavirus drugs effective against SARS-CoV-2 variants and future outbreaks.

2. Next-Gen Vaccines (Mucosal & Universal). Create intranasal vaccines for sterilizing immunity and variant-proof designs (e.g., nanoparticle/mosaic antigens).

3. Long COVID Mechanisms & Treatments. Uncover causes (viral persistence, autoimmunity?) and test targeted therapies (e.g., antivirals, immunomodulators).

4. Early Outpatient Therapies. Optimize antiviral combinations (e.g., Paxlovid + interferon) to prevent progression to severe disease.

5. Monoclonal Antibodies (mAbs) for Emerging Variants. Engineer broadly neutralizing mAbs resistant to viral escape mutations.

6. Immunomodulators for Severe COVID. Refine timing/dosing of steroids, JAK inhibitors, IL-6 blockers to curb cytokine storms without immunosuppression.

7. Biomarkers for Disease Severity. Identify predictive markers (genetic, cytokine profiles) to guide early intervention.

8. Antiviral Resistance Monitoring. Track emerging resistance to Paxlovid (nirmatrelvir), remdesivir, and molnupiravir.

9. Repurposed Drug Screening. Systematically test existing drugs (e.g., metformin, fluvoxamine) for antiviral/anti-inflammatory effects.

10. Rapid, Inexpensive Diagnostics. Develop at-home molecular tests (like rapid PCR) for early detection and treatment initiation.

11. Therapeutic Nanobodies. Advance inhaled nanobodies (smaller, cheaper than mAbs) for prophylaxis/treatment.

12. Combination Prophylaxis for High-Risk Groups. Test pre-exposure antiviral + antibody strategies in immunocompromised patients.

13. AI-Driven Drug Discovery. Use machine learning to accelerate identification of novel antivirals and immune targets.

14. Post-Exposure Vaccination Strategies. Determine if vaccination after exposure (like rabies PEP) reduces infection/severe outcomes.

15. Pediatric & Maternal COVID Treatments. Expand safety/efficacy data for antivirals, vaccines in pregnant women and children.

16. Global Access & Equity. Scale up generic antiviral production and low-cost vaccine platforms (e.g., protein subunit).

17. ACE2/TMPRSS2-Targeting Therapies. Develop entry inhibitors blocking viral fusion (e.g., camostat analogues, soluble ACE2 decoys).

18. Non-Spike Vaccine Targets. Explore conserved viral proteins (Nucleocapsid, M protein) for T-cell-focused immunity.

19. Nasal Antiviral Sprays. Test topical antivirals (e.g., nitric oxide, heparins) for prevention and early treatment.

20. Wastewater Surveillance & Early Warning. Improve real-time genomic sequencing of sewage to detect outbreaks/variants faster.

Perplexity

1. Pharmacological Treatments for Acute and Long COVID. Develop and test novel antivirals, immunomodulators, and combination therapies for acute COVID-19 and long COVID conditions, expanding beyond current options.
2. Vaccines: Efficacy, Safety, and Adaptation to Variants. Develop next-generation vaccines, improve effectiveness against emerging variants, optimize booster regimens, and ensure safety in all populations.

3. Early Diagnosis and Rapid Testing. Advance point-of-care, affordable diagnostics and early detection tools for acute infection as well as post-acute sequelae (long COVID).
4. Understanding the Pathophysiology of Long COVID. Uncover biological mechanisms underpinning persistent symptoms and multi-organ impact to guide targeted treatment.
5. Non-Pharmacological Symptom Management. Test and optimize rehabilitation, physical therapy, dietary interventions, and psychological support strategies.
6. Prevention of Transmission and Outbreak Control. Improve infection prevention strategies—vaccination, ventilation, mask innovations—and study societal compliance.
7. Immunity and Immune Response Research. Clarify natural and vaccine-induced immunity duration, immune correlates of protection, and immune dysfunction in long COVID.
8. Real-Time Genomic Surveillance and Variant Monitoring. Strengthen multi-source surveillance systems (including wastewater), global sharing of sequencing data, and rapid risk assessments for new variants.
9. Health System Strengthening and Scalability. Optimize models of scalable acute and post-acute (long COVID) care, triage, critical care capacity, and workforce readiness.
10. Equitable Access to Treatments and Vaccines. Address barriers to access in low-resource and vulnerable settings, including marginalized populations and global vaccine equity.
11. Comorbidity and Vulnerable Population Research. Tailor interventions and care models to those with high-risk comorbidities (cardiac, respiratory, metabolic) and special cohorts (elderly, immunocompromised, children).
12. Multidisciplinary Approaches to Mental Health. Address mental health sequelae—anxiety, depression, PTSD—among COVID-19 survivors, families, and healthcare workers.
13. Countering Misinformation and Risk Communication. Develop evidence-based strategies for public communication, vaccine confidence, and health-protective behaviors.
14. Socioeconomic and Health System Impact Studies. Analyze the socioeconomic consequences, disruptions to essential care, and impact on health inequalities—informing future pandemic preparedness.
15. Development of Predictive Biomarkers. Identify biomarkers for disease severity, prognosis, and response to treatments—supporting precision medicine approaches.
16. AI and Big Data Integration for Clinical Care. Harness artificial intelligence for diagnostics, risk stratification, treatment personalization, and real-time public health response.
17. Pediatrics and Pregnancy-Specific Research. Focus on maternal, fetal, and pediatric COVID-19 outcomes, treatments, vaccines, and management of unique sequelae.
18. Infection Prevention and Hospital Safety Innovations. Advance effective infection control within healthcare and congregate settings through new protocols and engineering solutions.

19. One Health and Zoonoses Research. Study animal-human-environment interfaces, origins of SARS-CoV-2, and future zoonotic threats to inform pandemic prevention.
20. Ethical, Legal, and Policy Considerations. Address ethical issues in research (consent, data privacy), resource allocation, and emergency policy responses for current and future pandemics.

B4: HIV

DeepSeek

1. Sterilizing & Functional Cures. Eradicate latent reservoirs via shock-and-kill (latency reversal + immune clearance), block-and-lock, or gene editing (CRISPR/Cas9).

2. Broadly Neutralizing Antibodies (bNAbs). Develop next-gen bNAbs (e.g., VRC07-523LS) for long-acting prevention/treatment and reservoir targeting.

3. Long-Acting Antiretrovirals (LA-ARVs). Improve injectable (e.g., lenacapavir, cabotegravir) and implantable formulations to replace daily ART.

4. HIV Vaccines. Advance mRNA-based, mosaic antigen, and germline-targeting vaccines to induce protective immunity.

5. Reservoir Mapping & Quantification. Refine assays (e.g., intact proviral DNA assay) to precisely measure latent HIV for cure trials.

6. Immune-Based Therapies. Harness CAR-T cells, checkpoint inhibitors, and TLR agonists to enhance immune control of HIV.

7. Gene Therapy & CRISPR. Edit CCR5/CXCR4 or excise HIV provirus using gene-editing tools (e.g., EBT-101 trial).

8. Targeting Viral Persistence Mechanisms. Block clonal expansion, HIV integration sites, and autophagy pathways that sustain reservoirs.

9. Paediatric HIV Cure Strategies. Optimize early ART initiation and immune-modulating therapies for perinatal infections.

10. Comorbidities & Aging with HIV. Address accelerated aging, neurocognitive decline, and cardiometabolic risks in PLWH.

11. Pre-Exposure Prophylaxis (PrEP) Innovations. Develop ultra-long-acting PrEP (e.g., 6-month injectables, vaginal rings) and on-demand oral PrEP.

12. ART-Free Remission Strategies. Test therapeutic vaccines + bNAbs to sustain viral control without daily drugs.

13. Drug Resistance Solutions. Design novel ARVs (e.g., capsid inhibitors, maturation inhibitors) for multidrug-resistant HIV.

14. Mucosal Immunity & Gut Repair. Restore gut barrier integrity and resident memory T-cells to reduce systemic inflammation.

15. Latency-Reversing Agents (LRAs). Improve HDAC inhibitors, PKC agonists, and BRD4 inhibitors to unmask hidden virus.

16. Nanotechnology for Drug Delivery. Engineer targeted nanoparticles to deliver ARVs/LRAs to sanctuary sites (e.g., brain, lymph nodes).

17. Economic & Implementation Research. Reduce costs of LA-ARVs/bNAbs and optimize "test-and-treat" strategies in low-resource settings.

18. Neuro-HIV Therapies. Develop CNS-penetrating drugs to combat HIV-associated neurocognitive disorders (HAND).

19. STI & HIV Co-Infection Management. Integrate MPOX, HCV, and STI prevention into HIV care programs.

20. Social Determinants & Equity. Tackle stigma, health disparities, and structural barriers to care access.

Perplexity

1. Achieving a Functional or Sterilizing Cure. Research targeting HIV latent reservoirs, including strategies to induce and eliminate (“shock and kill”), permanently silence (“block and lock”), or genetically remove HIV from infected cells.
2. Development of Next-Generation Antiretrovirals. Advancing safer, more effective, long-acting, and easier-to-administer ART regimens, such as injectables and implants.
3. Prevention of HIV Transmission. Creating effective HIV vaccines and optimizing pre-exposure prophylaxis (PrEP), including new options like injectable lenacapavir.
4. Understanding and Measuring HIV Reservoirs. Identifying, characterizing, and quantifying HIV reservoirs in the body, using novel imaging, assays, and biomarkers.
5. Alleviating Drug Resistance. Addressing mechanisms of ART resistance, monitoring resistance patterns, and developing strategies for those with multidrug-resistant HIV.
6. Pediatric and Adolescent Treatment Innovations. Developing optimal, age-appropriate ART formulations and cure strategies for infants, children, and adolescents.
7. Targeting Comorbidities and Co-infections. Understanding, preventing, and treating chronic diseases (e.g., cardiovascular, metabolic, liver) and infections (notably tuberculosis) in people living with HIV.
8. Long-term Safety and Toxicity Research. Investigating the long-term effects, side effects, and toxicity of ART, especially newer agents and long-acting formulations.
9. Minimizing Stigma and Improving Quality of Life. Reducing stigma, discrimination, and mental health burdens to facilitate ART adherence, retention, and holistic well-being.
10. Simplifying and Scaling Diagnostic Tools. Innovating rapid, affordable, and point-of-care diagnostics for viral load, resistance, and early infection suitable for low-resource settings.
11. Treatment Optimization in Special Populations. Customizing treatment for pregnant and breastfeeding women, co-infected patients, and individuals with co-morbidities.
12. Strategies for Sustained ART Adherence. Developing interventions to improve and support lifelong adherence, including digital health and peer-support models.
13. Implementation Science and Health Systems Research. Evaluating models of ART delivery, task-shifting, decentralization, and integrated care for scale-up and sustainability.

14. Behavioral, Social, and Ethical Research. Addressing behavioral barriers to prevention and treatment, and exploring ethical issues around HIV cure research, including community engagement.
15. Research Toward a Preventive or Therapeutic Vaccine. Advancing vaccine development for both prevention and post-infection control.
16. Real-World Outcomes and Data Analytics. Using “big data,” AI, and cohort studies to monitor outcomes, identify gaps in care, and optimize individualized approaches.
17. Harmonizing Global and Local Guidelines. Adapting and harmonizing ART and diagnostic guidelines for different epidemiologic and health system contexts.
18. Addressing Aging with HIV. Studying interactions between HIV, ART, and the aging process, including management of geriatric syndromes.
19. Community-Led Innovations. Empowering communities to shape research agendas, address barriers, and drive advocacy for equitable innovation uptake.
20. Cost-Effectiveness and Value-Based Research. Prioritizing resource allocation to maximize impact, ensure sustainability, and expand access to new treatments and diagnostics.

Part C: Texts for tweaked prompt analysis

C1: Alzheimer’s disease

DeepSeek

1. Early Detection & Biomarkers (Pre-Symptomatic Diagnosis). Why? Detecting AD before symptoms appear (via blood tests, CSF biomarkers, or PET imaging) allows earlier intervention when therapies may be most effective.

2. Disease-Modifying Therapies (Beyond Amyloid & Tau). Why? Most drugs target amyloid plaques, but mixed results suggest other pathways (neuroinflammation, synaptic dysfunction) need exploration.

3. Anti-Amyloid Strategies (Prevention & Clearance). Why? Despite controversies (e.g., aducanumab, lecanemab), amyloid remains a key target—better drugs with fewer side effects are needed.

4. Anti-Tau Therapies (Preventing Neurofibrillary Tangles). Why? Tau pathology correlates more closely with cognitive decline than amyloid; drugs blocking tau aggregation or spread are crucial.

5. Neuroinflammation & Microglial Modulation. Why? Chronic brain inflammation drives neurodegeneration; targeting microglia (e.g., TREM2 agonists) could slow progression.

6. APOE4 & Genetic Risk Factors (Therapeutic Targeting). Why? APOE4 is the strongest genetic risk factor—research into gene editing, lipid metabolism drugs, or APOE2-boosting therapies is vital.

7. Synaptic Repair & Neuroprotection. Why? Synapse loss precedes neuron death; drugs enhancing BDNF, NMDA modulation, or mitochondrial support may preserve cognition.

8. Blood-Brain Barrier (BBB) Penetration Strategies. Why? Many promising drugs fail due to poor BBB crossing; nanoparticle delivery or ultrasound techniques could improve efficacy.

9. Gut-Brain Axis & Microbiome Interventions. Why? Gut dysbiosis may worsen neuroinflammation; probiotics or fecal transplants could modify disease progression.

10. Lifestyle Interventions (Precision Prevention). Why? Diet (Mediterranean, ketogenic), exercise, and sleep optimization may delay onset—personalized approaches need validation.

11. Vascular Contributions (Treating Small Vessel Disease). Why? Hypertension and stroke risk factors exacerbate AD; managing vascular health may slow cognitive decline.

12. Alternative Proteinopathies (TDP-43, Alpha-Synuclein). Why? Many AD patients have mixed pathology; therapies targeting co-occurring proteins could improve outcomes.

13. Cognitive Reserve & Resilience Mechanisms. Why? Some individuals resist AD despite pathology; understanding protective factors (education, enrichment) could inspire therapies.

14. Digital Biomarkers (AI, Wearables for Early Detection. Why? Speech patterns, gait, or smartphone usage changes may predict AD earlier than traditional tests.

15. Sex Differences & Hormonal Therapies. Why? Women are disproportionately affected; estrogen’s role and sex-specific treatments need exploration.

16. Clinical Trial Design (Faster, More Inclusive Studies). Why? Trials are slow and costly; adaptive designs, better patient stratification, and decentralized trials could accelerate progress.

17. Combination Therapies (Multi-Target Approaches). Why? AD is multifactorial; combining amyloid/tau drugs with anti-inflammatories or metabolic regulators may be more effective.

18. Non-Invasive Brain Stimulation (tDCS, TMS). Why? Transcranial stimulation shows promise in enhancing cognition; optimal protocols for AD need development.

19. Prion-Like Spread of Misfolded Proteins. Why? Tau and amyloid may spread like prions; blocking this propagation could halt disease progression.

20. End-of-Life Care & Symptom Management. Why? Better palliative approaches for agitation, psychosis, and communication loss are needed for advanced AD.

Perplexity

1. Improved Early Detection and Diagnosis. Develop and validate novel biomarkers and digital tools to detect Alzheimer's disease (AD) and related dementias (ADRD) early and accurately in diverse populations.
2. Understanding Genetic and Molecular Mechanisms. Elucidate the underlying genetic, molecular, and cellular pathways driving AD onset and progression, including gene-environment interactions.
3. Development of Effective Disease-Modifying Therapies. Create therapies that can slow, halt, or reverse neurodegeneration, targeting amyloid, tau, inflammation, and other pathogenic processes.
4. Combination Therapies and Multi-Target Approaches. Advance strategies combining pharmacological and non-pharmacological interventions to address the multifactorial nature of AD.
5. Precision Medicine Approaches. Tailor prevention and treatment strategies based on individual risk profiles, genetic background, and disease subtype.
6. Addressing Health Disparities and Inclusion of Diverse Populations. Investigate differences in AD risk, progression, and treatment response across ethnic, racial, and socioeconomic groups to improve equity in care and research.
7. Lifestyle and Environmental Risk Modification. Study and implement interventions that mitigate risk factors such as cardiovascular health, diet, exercise, and social engagement.
8. Neuroinflammation and Immune System Modulation. Explore the role of inflammation and immune responses in AD pathology and use this knowledge to develop targeted therapies.
9. Advancement in Neuroimaging Technologies. Develop high-resolution, accessible imaging methods for better diagnosis, monitoring, and understanding of disease progression.
10. Development of Non-Pharmacological Interventions. Research effective cognitive, behavioral, and psychosocial therapies to improve quality of life for patients and caregivers.

11. Real-World Evidence and Clinical Research Methodology. Implement innovative clinical trial designs and use real-world data to accelerate translation of research into practice.
12. Understanding and Treating Co-Pathologies. Study common co-existing brain pathologies (vascular disease, Lewy bodies, TDP-43) and their impact on cognition and treatment outcomes.
13. Care and Support Systems Research. Innovate models for dementia care, including support for caregivers and integration of services to enhance wellbeing.
14. Public Awareness and Risk Communication. Improve communication strategies to raise awareness about AD risk factors, prevention, and available treatments.
15. Improving Cognitive Resilience and Brain Health. Identify biological and lifestyle factors that promote resilience to neurodegeneration to inform preventive strategies.
16. Expanding Brain Donation and Neuropathological Research. Increase participation in brain donation programs to correlate clinical and biological data for better understanding of AD.
17. Development of Digital Health Technologies. Leverage mobile and wearable devices for monitoring cognitive function and supporting patient care.
18. Exploration of Novel Therapeutic Targets. Investigate emerging biological pathways and targets beyond amyloid and tau proteins.
19. Ethical, Legal, and Social Implications Research. Study the broader impacts of AD advances on patients, families, and society, including privacy and consent issues.
20. Building Collaborative, Multidisciplinary Research Networks. Foster partnerships across academia, industry, and public health to accelerate discovery and application of treatments.

C2: Cancer

DeepSeek

1. Early Detection and Biomarker Development. Why? Early detection drastically improves survival rates. Research should focus on non-invasive biomarkers (liquid biopsies, imaging, AI-assisted diagnostics).

2. Immunotherapy Optimization. Why? Checkpoint inhibitors, CAR-T cells, and cancer vaccines show promise but need better response rates and reduced side effects.

3. Personalized and Precision Medicine. Why? Tailoring treatments based on genomics, proteomics, and tumor microenvironment will improve efficacy.

4. Overcoming Drug Resistance. Why? Resistance to chemotherapy, targeted therapy, and immunotherapy remains a major hurdle.

5. Targeting Cancer Stem Cells. Why? These cells drive recurrence and metastasis; eliminating them could cure some cancers.

6. Liquid Biopsies for Monitoring & Diagnosis. Why? Less invasive than tissue biopsies, allowing real-time tracking of tumor evolution.

7. Artificial Intelligence in Cancer Care. Why? AI can improve diagnostics, treatment selection, and predict patient outcomes.

8. Tumor Microenvironment Modulation. Why? The stroma, immune cells, and vasculature influence treatment resistance—targeting them could improve therapy.

9. Epigenetic Therapies. Why? Drugs targeting DNA methylation and histone modifications could reverse cancer-promoting changes.

10. Metastasis Prevention & Treatment. Why? >90% of cancer deaths are due to metastasis; understanding how to block it is critical.

11. Next-Gen Targeted Therapies (PROTACs, Degraders).Why? New drug modalities (e.g., protein degraders) could target "undruggable" oncoproteins.

12. Microbiome and Cancer Therapy Interactions. Why? Gut microbiota influences immunotherapy response and toxicity—manipulating it may improve outcomes.

13. Combination Therapies (Rational Drug Pairings). Why? Synergistic combinations could overcome resistance and improve survival.

14. Radiotherapy Enhancements (FLASH, Proton Therapy). Why? Advanced techniques could reduce side effects while improving tumor control.

15. Cancer Vaccines (Preventive & Therapeutic). Why? mRNA vaccines (like COVID tech) could train the immune system to attack cancer.

16. Nanotechnology for Drug Delivery. Why? Nanoparticles can improve drug targeting, reduce toxicity, and enhance efficacy.

17. Rare and Pediatric Cancers Research. Why? These cancers are understudied but devastating; new models and therapies are needed.

18. Cost-Effective & Accessible Therapies. Why? Many breakthroughs are expensive; research must focus on scalable, affordable solutions.

19. Mental Health & Survivorship Care. Why? Long-term side effects and psychological impact need better management strategies.

20. Global Disparities in Cancer Care. Why? Low-resource settings lack screening/treatment; research must address inequities.

Perplexity

1. Precision Oncology. Development and application of personalized treatments based on patients' genetic and molecular tumor profiles, aiming to maximize efficacy and minimize side effects.
2. Immunotherapy Development and Optimization. Advancing immune-based therapies (e.g., immune checkpoint inhibitors, CAR-T cells, cancer vaccines), including new targets and enhancement of response across different tumor types.
3. Early Detection and Diagnosis. Innovating technologies and tests such as liquid biopsies and multi-cancer early detection (MCED) assays for earlier, less invasive diagnosis, which directly improves survival rates.

4. Targeting Cancer Resistance and Recurrence. Understanding and overcoming mechanisms of treatment resistance (e.g., rare drug-tolerant cells, cancer stem cells) to improve durable responses and prevent relapse.
5. Biomarker Discovery and Validation. Identifying reliable biomarkers (molecular, immunological, imaging) for prognosis, prediction of therapy response, and patient stratification.
6. Artificial Intelligence (AI) and Data Science Integration. Applying AI/machine learning to pathology, radiology, genomics, and clinical data for improved diagnostics, prognostics, and personalized treatment planning.
7. Combination and Sequencing of Therapies. Designing rational combinations (immunotherapy, targeted therapies, conventional treatments) and optimal treatment sequences to enhance efficacy and minimize toxicity.
8. Reducing Cancer Disparities and Increasing Access. Research focused on addressing inequities in outcomes related to race, geography, socioeconomics, and improving cancer care delivery for underserved populations.
9. Tumor Microenvironment and Host Factors. Understanding how the surrounding non-cancer cells, immune components, and systemic factors influence tumor growth, response, and immune evasion.
10. Mechanisms of Metastasis and Disease Progression. Deciphering how cancers spread and transform into more aggressive, treatment-resistant forms to identify novel interventions.
11. Advancements in Minimally Invasive and Real-Time Monitoring. Refining liquid biopsies, synthetic biopsies, and circulating tumor DNA (ctDNA) analysis for dynamic and non-invasive treatment tracking.
12. Next-Generation Molecular and Cellular Therapies. Expanding cell-based therapies (e.g., novel CAR-T models, TIL therapies), gene editing, and oncolytic viruses in solid and hematologic cancers.
13. Cancer Prevention and Risk Reduction. Translational research into the interplay of genetics, environment, and behavior for effective prevention and risk stratification.
14. Therapeutic Cancer Vaccines. Developing and clinically validating vaccines that prevent or treat established cancers by stimulating targeted immune responses.
15. Understanding and Overcoming Toxicities and Side Effects. Designing interventions and predictive methods to minimize adverse effects while maintaining treatment potency (e.g., safer antibody-drug conjugate payloads).
16. Real-World Evidence and Implementation Science. Generating and applying real-world clinical data to close the gap between research findings and clinical practice, ensuring more rapid adoption of proven advances.
17. Pediatric and Rare Cancer Focus. Dedicated research to develop tailored therapies and improve outcomes in less-studied, high-need populations.
18. Surgical and Local Therapy Innovation. Enhancements in surgery, radiotherapy, and localized drug delivery to maximize tumor eradication and preserve normal tissue.
19. Cancer Survivorship and Quality of Life. Long-term care research addressing the physical, psychological, and social impacts of surviving cancer, including late toxicities and secondary cancers.

20. Patient and Public Involvement in Research. Ensuring research agendas and trial designs are shaped by patient experience for relevance and better health outcomes.

C3: COVID-19

DeepSeek

1. Universal Pan-Coronavirus Vaccines. Why? Current vaccines require frequent updates; a universal vaccine targeting conserved viral regions could provide long-term protection against all variants and future coronaviruses.

2. Antiviral Drugs for Early Treatment. Why? Drugs like Paxlovid reduce severe outcomes, but more affordable, broad-spectrum, and resistance-proof antivirals are needed.

3. Understanding & Treating Long COVID. Why? ~10-30% of survivors develop chronic symptoms (fatigue, cognitive impairment); research into mechanisms and treatments is urgent.

4. Next-Gen Monoclonal Antibodies (Resistant to Variants). Why? Many antibody therapies lose efficacy against new variants; engineered or multi-target antibodies could remain effective.

5. Nasal & Mucosal Vaccines (To Block Transmission). Why? Current vaccines reduce severe disease but don't fully prevent infection; mucosal immunity could stop transmission.

6. Predictors of Severe Disease (Biomarkers & AI Models). Why? Identifying high-risk patients early would allow targeted use of antivirals and monoclonal antibodies.

7. Broad-Spectrum Antivirals (Active Against Multiple Viruses). Why? Drugs like molnupiravir target SARS-CoV-2, but pan-viral drugs could prepare us for future outbreaks.

8. Immunity Durability & Boosting Strategies. Why? Waning immunity necessitates boosters; research should optimize timing and formulations for longer protection.

9. Variant-Proof Therapeutics (Small Molecules, Host-Targeting Drugs). Why? Unlike spike protein-targeting drugs, host-directed therapies (e.g., protease inhibitors) may resist viral mutations.

10. Long-Term Effects of COVID-19 on Organ Systems. Why? Beyond the lungs, COVID-19 affects the heart, brain, and kidneys; understanding mechanisms could guide treatments.

11. Improved Rapid Diagnostics (At-Home & Low-Cost). Why? Faster, cheaper, and more accurate tests would help control spread, especially in low-resource settings.

12. AI & Machine Learning for Outbreak Prediction. Why? Early detection of emerging variants and hotspots could guide public health responses.

13. Drug Repurposing for COVID-19 (Existing Medications). Why? Faster than developing new drugs; examples include fluvoxamine (anti-inflammatory) and colchicine.

14. Pediatric COVID-19 & MIS-C Treatments. Why? Children have unique risks (e.g., multisystem inflammatory syndrome); tailored therapies are needed.

15. Global Vaccine Equity & Delivery Systems. Why? Many countries still lack access; research into thermostable vaccines and last-mile delivery is critical.

16. Airborne Transmission Mitigation (Air Filtration, UV Tech). Why? Better indoor air quality standards could reduce superspreader events.

17. Combination Therapies (Antivirals + Immunomodulators). Why? Pairing antivirals with anti-inflammatories (e.g., dexamethasone) may improve outcomes.

18. Post-Vaccination Breakthrough Infections. Why? Research into why some vaccinated individuals still get severe COVID could improve vaccines.

19. Psychiatric & Neurological Impact of COVID-19. Why? Depression, anxiety, and "brain fog" are common; neuroprotective therapies may help.

20. Pandemic Preparedness (Lessons for Future Outbreaks). Why? COVID-19 exposed gaps in global response; research into surveillance, supply chains, and policy is vital.

Perplexity

1. Development of Effective Therapeutics. Continued innovation in antiviral drugs, host-directed therapies, and treatments that reduce severe disease and mortality.
2. Improved Vaccines and Vaccine Strategies. Creation of next-generation vaccines with broader variant coverage, longer-lasting immunity, and improved delivery methods.
3. Understanding Viral Variants and Evolution. Research into SARS-CoV-2 mutations, their impact on transmissibility, immune escape, and treatment resistance.
4. Long COVID and Post-Acute Sequelae Research. Elucidation of mechanisms, risk factors, and treatments for persistent symptoms following acute infection.
5. Rapid and Accurate Diagnostics. Development of sensitive, affordable, and accessible diagnostic tests for early detection and monitoring, including at-home options.
6. Host Immune Response and Pathogenesis. Detailed study of immune mechanisms in COVID-19—both protective and pathological—to inform therapies and vaccines.
7. Addressing Health Inequities. Strategies to ensure equitable access to prevention, diagnosis, and treatment worldwide, especially in low-resource settings.
8. Optimization of Clinical Management Protocols. Refinement of treatment guidelines for different COVID-19 severities and patient populations based on emerging evidence.
9. Therapeutic Use of Immunomodulators. Exploration of drugs modulating the immune response to prevent hyperinflammation and organ damage.
10. Antiviral Resistance Monitoring and Countermeasures. Surveillance for resistance to existing antiviral agents and development of strategies to overcome it.
11. Real-World Effectiveness and Safety Studies. Gathering evidence on how vaccines and treatments perform in varied populations and settings.
12. Transmission Dynamics and Infection Control. Understanding modes of transmission, including environmental and behavioral factors, to improve public health measures.

13. Use of Artificial Intelligence and Big Data Analytics. Leveraging AI to predict disease course, personalize treatments, and optimize resource allocation.
14. Integration of COVID-19 Care with Other Health Services. Improving care models that address COVID-19 alongside comorbidities and other infectious diseases.
15. Mental Health Impact and Support Interventions. Research on psychological consequences of COVID-19 and development of coping and treatment strategies.
16. Pediatric COVID-19 Research. Focus on disease impact, treatment, and vaccination specifically in children.
17. Global Health Preparedness and Rapid Response Systems. Building infrastructure and protocols for faster detection and containment of COVID-19 outbreaks and future pandemics.
18. Socioeconomic and Behavioral Research. Understanding how social factors and behaviors influence spread and outcomes, to guide policy and communication.
19. Innovations in Drug Delivery Systems. New delivery methods to improve treatment adherence and accessibility, such as long-acting injectables or inhaled therapies.
20. Sustainable Manufacturing and Distribution of Treatments. Ensuring scalable production and equitable distribution of vaccines and therapeutics globally.

C4: HIV

DeepSeek

1. HIV Cure Strategies (Sterilizing & Functional). Why? A cure remains the ultimate goal. Key approaches include: "Shock and kill" (latency reversal + immune clearance). Gene editing (CRISPR/Cas9 targeting HIV DNA). Immune-based therapies (CAR-T cells, broadly neutralizing antibodies)

2. Long-Acting Antiretrovirals (Injectable & Implantable ART). Why? Monthly or yearly injections (e.g., cabotegravir/rilpivirine) improve adherence and reduce transmission.

3. Broadly Neutralizing Antibodies (bNAbs) for Treatment & Prevention. Why? bNAbs can target multiple HIV strains and may replace or supplement ART.

4. HIV Vaccines (Preventive & Therapeutic). Why? Despite decades of research, no effective vaccine exists; mRNA technology (like COVID vaccines) offers new hope.

5. Targeting Viral Reservoirs (Latency Reversal & Elimination). Why? HIV hides in reservoirs (e.g., CD4+ T cells); eliminating them is key to a cure.

6. Pre-Exposure Prophylaxis (PrEP) Improvements. Why? Oral PrEP (e.g., Truvada) is effective but needs better global access, long-acting options (e.g., injectable cabotegravir).

7. Drug Resistance Mechanisms & New ART Classes. Why? Resistance threatens treatment efficacy; novel drugs (e.g., capsid inhibitors) are needed.

8. HIV & Aging (Managing Long-Term Complications). Why? People with HIV face accelerated aging, neurocognitive decline, and cardiovascular disease.

9. Pediatric HIV Cure & Better Treatments. Why? Children face unique challenges, including drug formulations and lifelong ART burdens.

10. HIV-Related Stigma & Mental Health Interventions. Why? Stigma reduces testing/treatment adherence; mental health support is critical.

11. CRISPR & Gene Editing for HIV Eradication. Why? Editing CCR5 (HIV co-receptor) or excising HIV DNA could lead to a cure.

12. Mucosal Immunity & Transmission Blockade. Why? Understanding mucosal HIV transmission could improve vaccines and microbicides.

13. Therapeutic Vaccines (Boosting Immune Control). Why? Could allow some patients to control HIV without daily ART ("functional cure").

14. Microbiome & HIV Progression. Why? Gut dysbiosis worsens inflammation; probiotics/fecal transplants may help.

15. Universal Access to ART & Health Equity. Why? 25% of people with HIV lack treatment access; cost, stigma, and infrastructure limit care.

16. HIV & Comorbidities (TB, Hepatitis, COVID-19). Why? Co-infections complicate treatment; integrated care models are needed.

17. Novel Drug Delivery Systems (Nanoparticles, Implants). Why? Could improve drug penetration into viral reservoirs (e.g., brain, lymph nodes).

18. Post-Treatment Control (Remission Without ART). Why? A small subset ("elite controllers") suppress HIV naturally—research could mimic this.

19. Women-Specific HIV Prevention & Treatment. Why? Biological/social factors increase women's vulnerability; better PrEP options needed.

20. HIV Cure Clinical Trial Design & Biomarkers. Why? Better biomarkers (e.g., viral reservoir assays) are needed to measure cure progress.

Perplexity

1. Reducing HIV Incidence. Focus on developing and optimizing safe and effective vaccines, microbicides, and pre-exposure prophylaxis (PrEP) to prevent new infections.
2. Development of Next-Generation HIV Therapies. Creating novel antiretroviral therapies (ART) that improve safety, ease of use (e.g., long-acting injectables), and overcome resistance.
3. Research Toward an HIV Cure. Innovations aiming to either eradicate HIV completely (sterilizing cure) or control it without ongoing treatment (functional cure), including targeting latent viral reservoirs.
4. Addressing HIV-Associated Comorbidities and Coinfections. Studying and managing diseases frequently occurring alongside HIV, such as tuberculosis, hepatitis, and non-communicable diseases.
5. Advancing Cross-Cutting Scientific Research. Integrating basic, behavioral, social, epidemiological, and implementation sciences to improve prevention, treatment, and care.
6. Optimization of Antiretroviral Treatment (ART). Improving drug regimens, dosing, and switching strategies to maximize efficacy and minimize side effects, including for special populations.

7. Development and Evaluation of Long-Acting Therapies. Research into injectables like lenacapavir and cabotegravir/rilpivirine to improve adherence and quality of life for people with HIV.
8. Improving HIV Diagnostics and Point-of-Care Testing. Enhanced diagnostics suitable for low- and middle-income settings, enabling rapid, accessible HIV testing and monitoring.
9. Pediatric and Adolescent HIV Treatment Research. Development of age-appropriate formulations and ensuring safety and efficacy data for children and adolescents.
10. Understanding and Targeting the Latent HIV Reservoir. Research into mechanisms maintaining viral latency and strategies to expose and eliminate dormant HIV-infected cells.
11. Prevention Science and Implementation. Addressing behavioral, structural, legal, and social barriers to prevention in diverse populations globally.
12. Addressing HIV Treatment Adherence and Retention. Developing interventions to support sustained adherence to ART and healthcare engagement.
13. Management of Drug Resistance. Surveillance and strategies to prevent and overcome resistance to current and future ART drugs.
14. Improving HIV Care Integration. Linking HIV services with mental health, non-communicable disease care, and sexual and reproductive health services.
15. Research on HIV in Key and Priority Populations. Focus on populations disproportionately affected, such as pregnant women, sex workers, men who have sex with men, and people who inject drugs.
16. Investigation of HIV-Associated Inflammation and Immune Dysfunction. Understanding HIV-induced chronic inflammation contributing to comorbidities despite viral suppression.
17. Innovations in HIV Cure Strategies. Exploration of novel approaches such as gene editing, immunotherapies, and latency-reversing agents.
18. Behavioral and Social Science Research. Understanding drivers of the epidemic and designing interventions to change behaviors and reduce stigma.
19. Real-World Evidence and Implementation Science. Using data from clinical practice to accelerate translation of research findings into effective policies and programs.
20. Strengthening Research Infrastructure and Capacity in Low- and Middle-Income Countries (LMICs). Building capacity for HIV research, clinical trials, and healthcare delivery in resource-limited settings to ensure equitable progress.
